# Interaction of bacteriophage P1 with an epiphytic *Pantoea agglomerans* strain—the role of the interplay between various mobilome elements

**DOI:** 10.3389/fmicb.2024.1356206

**Published:** 2024-03-25

**Authors:** Katarzyna Giermasińska-Buczek, Jan Gawor, Emil Stefańczyk, Urszula Gągała, Karolina Żuchniewicz, Hanna Rekosz-Burlaga, Robert Gromadka, Małgorzata Łobocka

**Affiliations:** ^1^Department of Biochemistry and Microbiology, Institute of Biology, Warsaw University of Life Sciences (SGGW-WULS), Warsaw, Poland; ^2^Institute of Biochemistry and Biophysics of the Polish Academy of Sciences, Warsaw, Poland

**Keywords:** bacteriophage P1, *Pantoea agglomerans* complete genome sequence, lysogeny, Tn*9* and mobile antibiotic resistance marker, plasmid curing, transduction, plasmid-prophage, replication and partition incompatibility

## Abstract

P1 is a model, temperate bacteriophage of the 94 kb genome. It can lysogenize representatives of the *Enterobacterales* order. In lysogens, it is maintained as a plasmid. We tested P1 interactions with the biocontrol *P. agglomerans* L15 strain to explore the utility of P1 in *P. agglomerans* genome engineering. A P1 derivative carrying the Tn*9* (cm^R^) transposon could transfer a plasmid from *Escherichia coli* to the L15 cells. The L15 cells infected with this derivative formed chloramphenicol-resistant colonies. They could grow in a liquid medium with chloramphenicol after adaptation and did not contain prophage P1 but the chromosomally inserted cm^R^ marker of P1 Tn*9* (*cat*). The insertions were accompanied by various rearrangements upstream of the Tn*9 cat* gene promoter and the loss of IS*1* (IS*1*L) from the corresponding region. Sequence analysis of the L15 strain genome revealed a chromosome and three plasmids of 0.58, 0.18, and 0.07 Mb. The largest and the smallest plasmid appeared to encode partition and replication incompatibility determinants similar to those of prophage P1, respectively. In the L15 derivatives cured of the largest plasmid, P1 with Tn*9* could not replace the smallest plasmid even if selected. However, it could replace the smallest and the largest plasmid of L15 if its Tn*9* IS*1*L sequence driving the Tn*9* mobility was inactivated or if it was enriched with an immobile kanamycin resistance marker. Moreover, it could develop lytically in the L15 derivatives cured of both these plasmids. Clearly, under conditions of selection for P1, the mobility of the P1 selective marker determines whether or not the incoming P1 can outcompete the incompatible L15 resident plasmids. Our results demonstrate that *P. agglomerans* can serve as a host for bacteriophage P1 and can be engineered with the help of this phage. They also provide an example of how antibiotics can modify the outcome of horizontal gene transfer in natural environments. Numerous plasmids of *Pantoea* strains appear to contain determinants of replication or partition incompatibility with P1. Therefore, P1 with an immobile selective marker may be a tool of choice in curing these strains from the respective plasmids to facilitate their functional analysis.

## 1 Introduction

Environmental bacteria offer a plethora of genes whose product, either by themselves or as parts of metabolic pathways, could find practical use in various fields of economics. The possibility of studying them in an isolated form, cloned in laboratory bacterial strains, is limited, often due to the association of their function with other functions or certain intracellular environments. Thus, the ability to knock out or modify the genes of interest directly in the cells of their natural hosts is important for elucidating their functions. This depends on the availability of genetic engineering tools that could be easily introduced to cells of environmental bacterial isolates, serving as sources of donor DNA for genetic exchanges or as platforms for expressing introduced genes. While plasmids are common tools in genetic manipulations of laboratory bacteria, their introduction to cells of environmental bacterial isolates is problematic mostly due to barriers from foreign DNA that are formed by the restriction-modification systems in addition to problems related to transformation (Dubnau, 1999; Ershova et al., 2015; Ren et al., 2019). Thus, there is a growing interest in the universal possibilities of foreign DNA introduction to environmental bacteria cells using wild-type or engineered bacteriophages (Westwater et al., 2002; Kittleson et al., 2012). One such bacteriophage is P1, which can productively infect or lysogenize various strains of *Enterobacterales* order and could even introduce heterologous DNA to bacterial cells that cannot be productively infected (Kaiser and Dworkin, 1975; Yarmolinsky and Sternberg, 1988; Łobocka and Gągała, 2021 and references therein; Giermasińska and Łobocka, 2016; Keller et al., 2021).

P1 is a temperate, tailed bacteriophage of myovirus morphology and 94 kb genome (Łobocka et al., 2004). It was isolated in 1951 by Giuseppe Bertani from the lysogenic *E. coli* strain Li and could infect a *Shigella desynteriae* strain (Bertani, 1951). Its transducing potential, discovered soon thereafter, became a basis for the wide use of P1 in the genetic mapping of *E. coli* chromosomes and the transfer of chromosomal genes between various *E. coli* strains (Lennox, 1955; Yarmolinsky and Sternberg, 1988). After infection, P1 can be maintained in a cell as a unit copy-number plasmid, and its mutants carrying antibiotic resistance markers facilitate the selection of lysogens (Konddo and Mitsuhashi, 1964; Goldberg et al., 1974). The plasmidial form of prophage P1 allows one to combine the high efficiency of heterologous DNA introduction to bacteria through infection with the possibility of studying the expression of cloned genes. The P1 head can accommodate ~112% of the P1 genomic DNA. This 12% of redundant information can be potentially replaced by heterologous DNA, even by a whole plasmid, without interfering with phage and prophage functions if inserted in a non-essential genome region. Another possibility is the construction of P1-based phagemids—plasmids that additionally contain the P1 lytic replicon and P1 *pac* site to initiate their packaging to P1 phage heads if other required functions are provided from the P1 helper phage (Westwater et al., 2002). Therefore, P1 has practical use in transferring heterologous plasmids to bacterial cells and is a vector for heterologous DNA expression by itself. The latter possibility has been facilitated by the recent development of simple and traceless methods of targeted P1 DNA mutagenesis, including the introduction of foreign genes of unknown phenotypes to P1 (Bednarek et al., 2023).

The entering and maintenance of P1 lysogeny is controlled by the P1-encoded C1 protein—a repressor of lytic functions (reviewed in Yarmolinsky, 2004). The introduction to a common use of P1 *c1-100*, a P1 mutant which encodes a temperature-sensitive C1 protein, allows the synchronous induction of P1 lytic development in lysogens by a shift in the growth temperature of the lysogen culture (Rosner, 1972). The stable inheritance of the P1 plasmid-prophage in a population depends on the synchronization of plasmid replication with the cell cycle, on the tight control of the P1 plasmid copy number, on the resolution of plasmid multimers to monomers, and on the active segregation (partition) of newly replicated plasmid copies to the centers of future daughter cells before cell division (Łobocka et al., 2004). The replicon of P1 plasmid-prophage belongs to the IncY replicons (Yarmolinsky and Sternberg, 1988). Its replication initiator protein, RepA, controls the replication initiation and copy number of the P1 plasmid-prophage by binding to two groups of 19 bp sequences (iterons) upstream and downstream of the *repA* gene (Chattoraj and Schneider, 1997). The iterons also determine the replication incompatibility of plasmid-prophage P1. The active partition of P1 depends on an operon of two genes, *parAB*, which ends with the *parS* site—an analog of eukaryotic centromere serving as an anchor for partitioning machinery through binding of ParB protein (Surtees and Funnell, 2003). Plasmids with similar partition systems and conserved ParB-*parS* interaction sites are incompatible with prophage P1.

P1 was shown to infect and lysogenize certain Gram-negative bacteria representing mainly *Enterobacteriaceae* and *Rhizobiaceae* families (Yarmolinsky and Sternberg, 1988; Łobocka et al., 2004; Giermasińska and Łobocka, 2016; Łobocka and Gągała, 2021; Bednarek et al., 2022). It indicates its natural adaptation to taxonomically different hosts. Certain P1 properties contributing to its wide host range have been identified. P1 adsorption to bacteria depends on six kinked tail fibers. The invertible C-segment of P1 DNA encodes two alternative variants of the C-terminal parts of tail fibers with different specificities. The variant, which is predominantly expressed in *E. coli* K-12 cells, has been suggested to adsorb to the terminal glucose of the LPS outer core oligosaccharide. The phages with the oppositely oriented C-segment could adsorb to *Shigella flexneri* 2a 2457O, *S. flexneri* 5a M90T, and *E. coli* O3 strain cells (Huan et al., 2022). The injected phage DNA is protected from degradation by restrictases of different subsets of enterobacterial type I restriction-modification systems by two P1 head proteins, DarA and DarB (Walker and Walker, 1981; Iida et al., 1987; Streiff et al., 1987). The multimer resolution system composed of recombinase Cre and its site of action, *loxP*, was functional in different cellular environments, including eukaryotic cells (reviewed by Yarmolinsky and Hoess, 2015). The lysis of taxonomically different host cells by P1 at the release of phage progeny is ensured by an unusually complex system of P1 cell lysis genes (Bednarek et al., 2022).

*Pantoea agglomerans*, a motile, yellow-pigmented rod of the *Enterobacteriaceae* family, is widely distributed in nature (reviewed by Walterson and Stavrinides, 2015). Its name was assigned in 1989 to bacteria known formerly as *Enterobacter agglomerans, Erwinia herbicola*, and *Erwinia millatiae. P. agglomerans* strains have been isolated from various aquatic and terrestrial environments, humans, animals, and plants. They can often be found among plant endophytes or epiphytes, playing a beneficial bioprotective role, but some of them are plant pathogens causing galls, wilting, soft rot, and necrosis in a variety of agriculturally relevant plants (Lindow et al., 1998; Walterson and Stavrinides, 2015). Certain strains of *P. agglomerans* inhibit the growth of bacteria or fungi pathogenic to plants by competition, production of antibiotics, cell wall-degrading enzymes, siderophores, hydrogen cyanide, and by the induction of systemic resistance (Wilson and Lindow, 1994; Thissera et al., 2020; Carobbi et al., 2022; Xu et al., 2022; Matilla et al., 2023; reviewed by Lorenzi et al., 2022). Phylogenetic analysis based on the sequences of 16S rDNA and *fusA, gyrB, leuS, pyrG, rplB*, and *rpoB* genes revealed a high similarity between *P. agglomerans* and *E. coli*—a hallmark of common ancestry of these two bacterial species (Delétoile et al., 2009). This prompted us to test whether bacteriophage P1 could be used as a molecular biology tool in studies on *P. agglomerans* L15 (an environmental epiphytic isolate of *P. agglomerans*), which has shown to be antagonistic to certain fungal plant pathogens (Rekosz-Burlaga et al., 2014).

## 2 Materials and methods

### 2.1 Bacterial strains, plasmids, bacteriophages, and oligonucleotides

*E. coli* strains N99 (*galK2 strA*; Cole and Guest, 1980), C600 (*F- supE44 hsdR17 thi-1 thr-1 leuB6 lacY1 tonA21*; Sambrook et al., 1989), and DJ125 (*recA*^−^ derivative of C600; Łobocka and Yarmolinsky, 1996) were used for phage propagation, lysogenization, transformation, cloning and plasmid isolation experiments, where indicated. *Pantoea agglomerans* L15 is an epiphytic strain with biocontrol properties isolated from *Hypericum perforatum* leaves (Rekosz-Burlaga et al., 2014). Its derivatives deprived of one or two plasmids were constructed in this work, as described further in the text. P1 *c1-100* Tn*9* (Łobocka et al., 2004), a mutant of P1 bacteriophage, was used to study the interaction with *P. agglomerans* L15. It carries the Tn*9* transposon conferring the chloramphenicol resistance to its lysogens. Additionally, the *c1-100* mutation makes the repressor of its lytic functions thermosensitive, enabling the synchronous thermal induction of P1 lytic development in lysogens. The P1 *c1-100* Tn*9* derivatives, with the IS*1* sequence, inactivated by the insertion of kanamycin resistance cassette (P1 *c1*-*100* IS*1*::km^R^) or the *ccdA* gene of pPagL15_3 plasmid (P1 *c1*-*100* IS*1*::*ccdA*_pPagL15_3_), were constructed by replacing wild-type IS*1* with inactivated IS*1* using homologous recombination and plasmid pKGI10 and pKGI12, respectively, as donors of the inactivated IS*1*. The replacement method has been described previously (Bednarek et al., 2023). Plasmids that were used in this study are listed in Supplementary Table 1. The sequence correctness of DNA fragments obtained by PCR amplification and cloned in plasmids was verified by DNA sequencing with appropriate primers. The oligonucleotides used for the DNA amplification, construction of plasmids, or verification of plasmid sequence correctness are listed in Supplementary Table 2. DNA sequencing and oligonucleotide synthesis were performed at the Laboratory of DNA Sequencing and Oligonucleotides Synthesis of the Institute of Biochemistry and Biophysics of the Polish Academy of Sciences.

### 2.2 Media and bacterial growth conditions

Bacteria were grown in LB liquid medium with aeration or on LA solid medium (LB with 1.5% agar) at 30, 33, 35, 37, or 42°C, where indicated (Miller, 1972). Solid minimal M9 medium supplemented with glucose (0.2%) with or without thiamine (1 μg/mL) was used in certain experiments (Miller, 1972). Antibiotics were added to liquid or solid media, when appropriate, at the following final concentrations: ampicillin −50 or 100 μg/mL, chloramphenicol −12.5 μg/mL, kanamycin −12.5 or 25 μg/mL, and tetracycline (10 μg/mL). Bacterial growth was monitored by measuring the optical density of cultures at 600 nm (OD_600_). Media used for phage adsorption or infection experiments were supplemented with CaCl_2_ and MgSO_4_ (to the final concentration of 5 mM each).

### 2.3 Induction of phage lytic development in lysogens, and phage propagation

Overnight cultures of lysogens grown at 30°C in LB medium supplemented with chloramphenicol or kanamycin (12.5 μg/mL each), where indicated, were diluted 50 times in a similar medium supplemented with glucose (0.2%) and grown with shaking (200 RPM/min) until the optical density OD_600_ of about 0.4 (*E. coli* lysogens) or 0.5 (*P. agglomerans* lysogens). Prophage induction was initiated by the rapid heating of cultures to 43°C. The warmed-up cultures were incubated at 42°C with shaking for 30 min (*E. coli*) or 15 min (*P. agglomerans*), transferred to 37°C (*E. coli*) or 33°C (*P. agglomerans*), and incubated with shaking until the signs of lysis could be seen (clearing *E. coli* culture or slightly visible flocculation of *P. agglomerans* culture). In the case of *E. coli*, lysis typically occurred in about 50 min from induction, while in the case of *P. agglomerans*, the signs of flocculation were visible in about 90 min from induction.

Bacteriophages were propagated upon thermal induction of lysogens, as described previously (Bednarek et al., 2022). Briefly, *E. coli* cells lysogenized with P1 *c1*-*100* IS*1*::Tn*9* or its mutant derivatives were grown overnight in LB supplemented with chloramphenicol (12.5 μg/mL) at 30°C. The overnight cultures were diluted 50 times in fresh LB with 0.2% glucose and chloramphenicol or kanamycin, where indicated, and grown at 30°C until the optical density (OD_600_) reached 0.3–0.4. Prophages were thermally induced by rapid heating of cultures to 42°C. Then, the cultures were returned to 42°C and grown with shaking for about 45–60 min until the signs of lysis were manifested. When the OD600 of lysed cultures fell to 0.15 they were centrifuged at 29,030 × g for 40 min at 4°C to pellet bacteria. The remaining cell remnants were removed from the lysates by filtration through a 0.22 μm syringe filter (Filtropur, Sarstedt, Nümbrecht, Germany). The titer of phages in the lysates was assayed using the standard double-layer agar method, according to Adams (1959).

### 2.4 Lysogenization

Portions (100 μL) of overnight cultures of *E. coli* N99 or *P. agglomerans* L15 grown in LB medium at 30°C with aeration, supplemented with CaCl_2_ and MgSO_4_, were gently mixed with 100 μL of appropriately diluted lysate containing phages to MOI ≤ 1, and incubated for 25–30 min at room temperature without shaking. Following that, free calcium ions were removed from the mixtures by adding 200 μL 0.5 M sodium citrate to block the adsorption of the remaining phages. The mixtures were supplemented with 1 mL of LB medium, incubated for 1 h at 30°C to express the antibiotic resistance gene, and plated on LA medium containing chloramphenicol (12.5 μg/mL) or kanamycin (25 μg/mL), where indicated. Plates were incubated for ~36 h at 30°C. Each experiment was repeated three times.

### 2.5 Plasmid transduction

Overnight cultures of *E. coli* C600 cells grown at 30°C in LB medium were refreshed by 50 times dilution in a similar medium and grown with shaking (200 RPM/min) until the optical density (OD_600_) of about 0.3 or 1.4, where indicated. Samples (0.1 mL) withdrawn from the cultures were supplemented with 0.1 mL of lysate with phages (10^5^-10^8^ pfu/mL, where indicated), 0.1 mL of CaCl_2_ and 0.1 mL MgSO_4_ (20 mM each), gently mixed and incubated at room temperature without shaking for 25–30 min. Next, 0.2 mL of 0.5 M sodium citrate was added to each mixture to stop phage adsorption. The mixtures were supplemented with 0.5 mL LB medium and incubated at 30°C with shaking for 1 h to express the plasmid antibiotic resistance marker. Equal aliquots of each mixture were plated on an LA medium with chloramphenicol, tetracycline, or chloramphenicol and tetracycline. The transduction procedure of *P. agglomerans* L15 cells was performed using 10 times increased volumes of all components of the transduction mixture. After adding the LB medium to the mixture, the mixtures were incubated overnight at room temperature, centrifuged to pellet the bacteria, and resuspended in 300 μL of LB medium. Equal aliquots of the suspensions were plated on the LA medium with chloramphenicol, tetracycline or chloramphenicol, and tetracycline. The plated bacteria were incubated for about 36 h at 30°C. The acquisition of P1 transducing phages and optimization of P1 mediated plasmid transduction procedure are described in detail in the Supplementary material.

### 2.6 Testing the stability of chloramphenicol resistance carrier

The stability of the chloramphenicol resistance carrier was tested as described previously by Grigoriev and Lobocka (2001). Briefly, the colonies of *P. agglomerans* L15 that were obtained after infection with P1 *c1-100* Tn*9* as grown on LA medium with chloramphenicol were purified once by restreaking on the same medium and restreaked again on LA medium without chloramphenicol. Three colonies of each clone were cut out with a portion of underlying agar, resuspended in 1 mL of LB, and vortexed vigorously. One hundred μL portions of serially diluted cell suspensions were plated on LB plates without chloramphenicol and incubated for ~36 h at 30°C. Grown colonies were replica plated on fresh LA medium with and without chloramphenicol. The comparisons of colony counts on plates with and without chloramphenicol were used to calculate the fraction of the cell population that retained the chloramphenicol resistance marker. Calculations were based on colony counts from the progeny of verified carriers of the chloramphenicol resistance marker. Verification was based on chloramphenicol-resistant colony-forming units in undiluted cell suspensions.

### 2.7 Mapping of the Tn*9* transposon insertion sites

**Figure 1:**
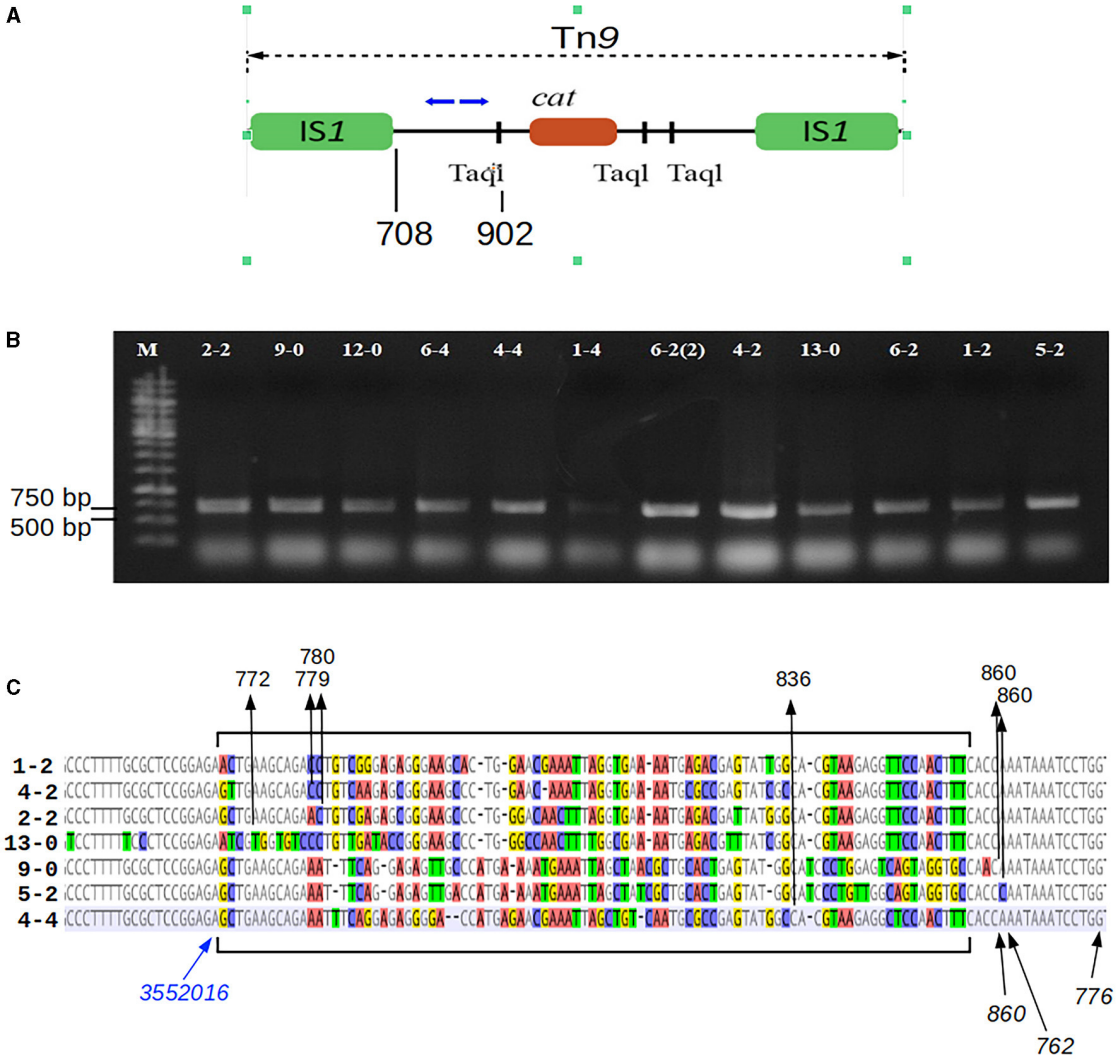
Mapping of the Tn*9* insertion sites in the P1-infected *P. agglomerans* L15 clones that acquired the resistance to chloramphenicol. **(A)** The mapping was performed by sequencing circular ligation products obtained after the digestion of the total DNA of the cmR-resistant L15 clones with TaqI. Blue arrows above the scheme of Tn*9* show the upper (OMLO739) and lower (OMLO738 or OMLO763) primers used for amplification and sequencing. Positions of the right end of left IS*1* and the leftmost TaqI site are indicated below the scheme. **(B)** Amplicons of 12 independently isolated cm^R^ clones. **(C)** Alignment of the border regions between the sequence of the L15 chromosome and the Tn*9*-derived DNA at the insertion sites of Tn*9* with different insertion sites. The differentiated fragments of the border regions are bracketed. The black arrows with numbers at the top of the alignment indicate the junctions between L15- and Tn9-derived DNA. The numbers represent the coordinates of Tn*9*-derived DNA in the complete sequence of Tn*9*. The blue arrow indicates the leftmost coordinate of the L15 genomic sequence that is common to all clones (see GenBank acc. no. CP034148).

The DNA of bacteriophage P1 *c1-100* Tn*9* or the total genomic DNA *P. agglomerans* cells obtained as stable chloramphenicol-resistant clones following the infection with P1 *c1-100* Tn*9* were digested with restriction endonuclease Taq1, which recognizes three sites within the Tn*9* transposon, between the IS*1* sequences of Tn*9*. One of these sites is between the left IS*1* (IS*1*L) and the beginning of the *cat* gene. The two other sites are between the end of the *cat* gene and the right IS*1* (IS*1*R) of Tn*9* (Figure 1). The digested DNA was diluted 20 times to increase the probability of preferential encounters of ends of the same fragment. The fragments were circularized by ligation with the T4 phage DNA ligase. To identify the borders between the *P. agglomerans* L15 DNA and the left end of Tn*9* in the Tn*9* insertion sites, we used the ligation mixture as a source of templates in PCR amplification with primers OMLO738 and OMLO739 specific for the Tn*9* sequence between the IS*1*L and the leftmost TaqI recognition site and oriented outwards. The products of the PCR amplification were separated electrophoretically in 1.0% agarose gel, cut out of the gel, and purified using the Gel Out kit (A&A Biotechnology, Gdańsk, Poland). The purified DNA fragments were amplified again with OMLO738 and OMLO739 primers, separated again in 1.0% agarose gel, purified from the gel as described above, and sequenced with the use of the same primers. In the case of too short sequence reads, they were sequenced additionally with OMLO763 primer.

### 2.8 DNA isolation and manipulation

Total DNA was isolated from bacteria using a Genomic Mini kit (A&A Biotechnology, Gdansk, Poland) according to the manufacturer's recommendations. Plasmid DNA was isolated from cells with the use of Qiagen Plasmid Midi kit (Qiagen, Inc., Chatsworth, Calif.) or Plasmid Mini kit (A&A Biotechnology, Gdańsk, Poland) as recommended by the kit providers. Restriction digestions, ligations, and DNA amplifications were performed according to the standard protocols (Sambrook et al., 1989) or as recommended by enzyme suppliers. DNA was analyzed by electrophoresis in agarose gels as described previously (Sambrook et al., 1989).

### 2.9 *P. agglomerans* L15 genomic DNA sequencing and sequence assembly

The determination of the total *P. agglomerans* L15 DNA sequence was performed at the Laboratory of DNA Sequencing and Oligonucleotide Synthesis of the Institute of Biochemistry and Biophysics, Polish Academy of Sciences (Warsaw, Poland), by whole genome sequencing (WGS). Bacterial total genomic DNA was isolated using the CTAB/lysozyme method (Wilson, 2001). DNA quality was checked on standard 0.8% agarose gel, and 1% PFGE gel on Biorad CHEF-III apparatus (BioRad, Hercules, USA). The template quantity was measured using a Qubit fluorimeter (Thermo Fisher Scientific, Waltham, USA). The genomic bacterial DNA was mechanically sheared to the appropriate size and used for Paired-End TruSeq-like library construction using the KAPA Library preparation kit (KAPA/Roche, Basel, Switzerland) following the manufacturer's instructions. The bacterial genome was sequenced in paired-end mode (v3, 600 cycle chemistry kit) using the MiSeq instrument (Illumina, San Diego, CA). Illumina sequencing yielded 804,080 reads and 1,053,625,110 nucleotides of sequence data. The obtained sequence reads were filtered by quality using the FastX toolkit (http://hannonlab.cshl.edu/fastx_toolkit/), and residual Illumina adapters were removed using Cutadapt (https://github.com/marcelm/cutadapt). Quality-filtered Illumina data (1,204,850 paired reads and 357,606,583 nucleotides of sequence data) were assembled using Spades v3.8.0 (http://cab.spbu.ru/software/spades/) to estimate the approximate size of the draft bacterial genome. In the next stage, long reads were generated using the MinION nanopore sequencing instrument (Oxford Nanopore Technologies, Oxford, UK). Bacterial DNA was sheared into ~10 kb fragments using Covaris gTube (Covaris, Ltd., Brighton, United Kingdom), and the library was constructed using the ONT 1D ligation sequencing kit (SQK-LSK108) with the native barcoding expansion kit (EXP-NBD103). Nanopore sequencing was performed using the NC_48 h_Sequencing_Run_FLO-MIN106_SQK-LSK108 protocol and R9.4 MinION flowcell. Raw nanopore data were basecalled using Albacore v2.1.7 (Oxford Nanopore Technologies, Oxford, UK). After quality filtering and sequencing adapter removal using Porechop (https://github.com/rrwick/Porechop), 20,701 barcoded reads remained. The obtained dataset was quality-checked using NanoPlot (De Coster et al., 2018). The median read length of the obtained dataset was 7,736 nucleotides and 164,707,659 of total bases. Long nanopore reads were assembled in a hybrid mode with Illumina data using Spades v3.8.0. Genome hybrid assembly resulted in four circular replicons: a 4 Mb size chromosome and three plasmids. The remaining sequence errors in the genome assembly were verified by the PCR amplification of DNA fragments, followed by Sanger sequencing with an ABI3730xl Genetic Analyzer (Life Technologies, USA) using BigDye Terminator Mix v. 3.1 chemistry (Life Technologies, USA). All sequence errors and misassemblies were further corrected using the Seqman software (DNAStar, USA) to obtain the complete nucleotide sequence of the bacterial genome.

### 2.10 DNA sequence analysis and annotations

Protein coding genes in the assembled DNA sequences were annotated automatically with the use of NCBI Prokaryotic Genome Annotation Pipeline (released 2013) https://www.ncbi.nlm.nih.gov/genome/annotation_prok/ (Tatusova et al., 2016; Haft et al., 2018). Additional protein-coding genes were identified with the help of the RAST annotation server (Aziz et al., 2008; Overbeek et al., 2014; Brettin et al., 2015). The annotations were corrected manually, using the results of the comparative analysis of predicted proteins with proteins in the NCBI database of non-redundant protein sequences (NR) with Blastp (Altschul et al., 1997). A search for conserved sequence motifs in selected proteins was done by comparing each protein sequence with motifs in the InterProScan [http://www.ebi.ac.uk/Tools/pfa/iprscan5/] (Jones et al., 2014) protein sequence motifs library. BLASTn (https://blast.ncbi.nlm.nih.gov/Blast.cgi, accessed on 29 June 2023) was used to find the closest relatives of the tested *P. agglomerans* strains and its plasmids among *Pantoea* strains and plasmids of completely sequenced genomes deposited in GenBank. A scheme of the plasmid genome organization was made using the Proksee package (Grant et al., 2023). A search for the presence of homologs of essential *E. coli* K-12 substr. MG1655 proteins among the predicted proteins of *P. agglomerans* L15 was performed using tblastx and the database of essential *E. coli* K-12 substr. MG1655 proteins at https://shigen.nig.ac.jp/ecoli/pec/ (Hashimoto et al., 2005; Kato and Hashimoto, 2007; Yamazaki et al., 2008).

To detect potential anti-phage systems in the *P. agglomerans* chromosome (NZ_CP034148) and plasmids (NZ_CP034149, NZ_CP034150, and NZ_CP034151), the DefenseFinder tool was used with the database version 1.2.2 and default settings (Abby et al., 2014; Tesson et al., 2022). Additionally, the predicted products of sequenced DNA molecules were searched manually for the presence of proteins similar to those of known anti-phage defense systems of confirmed activity against bacteriophage P1.

## 3 Results

### 3.1 Bacteriophage P1 *c1-100* Tn*9*-mediated plasmid transfer from *E. coli* to cells of an epiphytic *Pantoea agglomerans* isolate

We tested whether the commonly used P1 *c1-100* Tn*9* mutant, which is thermo-inducible and carries the chloramphenicol resistance marker in the Tn*9* transposon, could be used as a convenient vector in foreign DNA transfer by transduction to epiphytic *Pantoea agglomerans* strain L15 isolated from *Hypericum perforatum* leaves (Rekosz-Burlaga et al., 2014). The adsorption of P1 *c1-100* IS*1*::Tn*9* to cells of the L15 strain was indistinguishable from that to cells of the *E. coli* N99 strain, which served as a phage propagation host (Supplementary Figure 1), suggesting the sensitivity of *P. agglomerans* L15 to infection with P1. To verify whether P1 *c1-100* Tn*9* can transfer foreign plasmid DNA to *P. agglomerans* L15 cells, we used it to infect *E. coli* cells carrying the wide-host-range pRK2 (tc^R^) plasmid derivatives with cloned fragments of the P1 genome to provide the regions of homology with prophage P1 (see Supplementary Table 1). Colonies of infected cells that could grow on chloramphenicol and tetracycline were used to induce P1 lytic development and to obtain plasmid-transducing phages (see subchapter 1.2 of Supplementary material). Of the tested plasmids, the pKGI5 plasmid containing the cloned fragment of P1 *phd doc* operon could be transferred by P1 *c1-100* Tn*9* between *E. coli* cells (Supplementary Table 3, Supplementary Figure 2), and also from *E. coli* to *P. agglomerans* L15 cells, albeit less efficiently (Supplementary Table 4, Supplementary Figures 3, 4). This shows that P1 can be a vector in DNA transfer between these two bacterial species.

### 3.2 Bacteriophage P1 *c1-100* Tn*9*-mediated transfer of chloramphenicol resistance gene to cells of *P. agglomerans* L15

When the L15 cells were treated with P1 *c1-100* IS*1*::Tn*9* to obtain lysogens and plated on LA medium with chloramphenicol, colonies appeared on this medium after overnight incubation at 30°C. They could be restreaked on a similar medium with chloramphenicol, where also they formed colonies. However, when these colonies were used to inoculate liquid medium with chloramphenicol, most of their overnight cultures did not reach the optical density (OD_600_) of over 0.05. The refreshment of such cultures (1:50) in a fresh medium with chloramphenicol and their prolonged incubation in 19 of 40 cases resulted in growth improvement, suggesting the stabilization of the chloramphenicol resistance phenotype. Besides, when these cultures were diluted and plated on media with and without chloramphenicol, the number of single colonies obtained was similar on both kinds of media. However, our attempts to thermally induce lytic development of P1 *c1-100* Tn*9* in these potential lysogens failed. Further, while the total DNA isolated from them could serve as a template to obtain the amplicons with a primer pair specific for the *cat* gene of Tn*9* transposon, we did not obtain any amplicons with a primer pair specific for a region of P1 DNA outside of the Tn*9* insertion (Supplementary Figure 5). This suggested that P1 infected cells were not lysogenized with P1 but only acquired the chloramphenicol resistance marker of P1 Tn*9*.

### 3.3 Mapping of the Tn*9* insertion in the chloramphenicol-resistant derivatives of P1 *c1-100* Tn*9*-treated *P. agglomerans* L15 cells

To identify the sites of Tn*9* transposition from the DNA of phage P1 *c1-100* Tn*9* to the *P. agglomerans* L15 genome, we mapped the insertion of Tn*9* in nine independently selected stable chloramphenicol-resistant L15 clones obtained after infection with P1 *c1-100* Tn*9* (Figure 1, see Materials and methods section). The *Pantoea*-derived sequences at the border of Tn*9* in all clones represented the regulatory and 5′ region of a gene encoding a DUF1471 domain-containing protein of unknown function (WP_013359194.1, see further in this manuscript). However, the insertion sites were not the same, varying up to 82 nucleotide residues downstream from the last L15 chromosome coordinate common to all clones (Figure 1C). Further, the Tn*9-*derived sequences at the border differed between the clones by up to 79 nucleotide residues. Moreover, most represented the rearranged fragments of the region between the leftmost TaqI site and IS*1*L, but none included IS*1*L. Clearly, the insertion of Tn*9* in its target site was associated with the deletion of IS*1*L and the accompanying rearrangements and deletions in the Tn*9* sequences to the right of IS*1*L (Figure 1C). The deletion was most likely associated with IS*1*L transposition to a different genomic region. Positive results of PCR amplification with primers complementary to the internal region of IS*1* (OMLO759 and OML760) and the DNA of the tested clones indicate that they contain at least one IS*1* sequence (data not shown). The loss of IS*1*L of Tn*9* in the L15 clones must have occurred during or after the transfer of Tn*9* from the genome of P1 *c1-100* Tn*9* to the genome of L15. The sequence analysis of circularized amplicons of TaqI-cleaved P1 *c1-100* Tn*9* DNA with the primers that were used for Tn*9* mapping in the L15 chromosome confirmed the results of De Bruijn and Bukhari (1978) showing that Tn*9* in P1 *c1-100* Tn*9* is inserted in the native IS*1* sequence of P1 and contains IS*1*L (data not shown).

### 3.4 Identification of the *P. agglomerans* L15 traits that could be potentially responsible for the inability of P1 *c1-100* Tn*9* to stably lysogenize this strain

**Figure 2:**
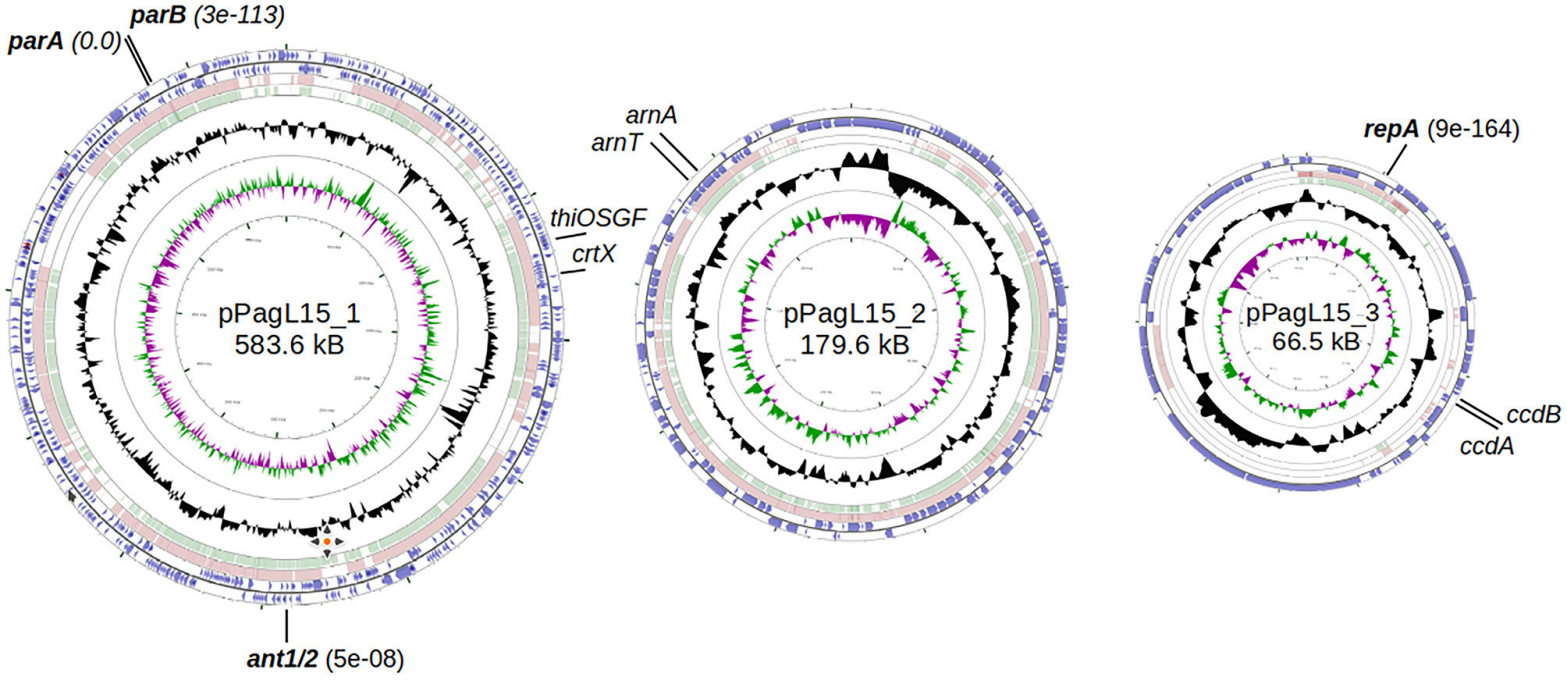
Genome maps of *P. agglomerans* L15 plasmids: pPagL15_1, pPAGL15_2, and pPagL15_3 (GenBank acc. no. CP034149, CP034150, and CP034151). The two outermost circles in each map represent genes transcribed in clockwise and counterclockwise directions, respectively. The two innermost circles show GC content and G-C skew. Circles in between show regions similar in sequence to those of the two closest representatives of each plasmid in the GenBank: CP016890 and CP001895, CP016892 and CP001893, and CP016891 and CP001894 in the case of pPagL15_1, pPAGL15_2, and pPagL15_3, respectively. Plasmid genes whose products encode close homologs of P1 proteins that may be responsible for the inability of P1 to stably lysogenize the L15 strain are in bold. Other genes indicated were used in curing the L15 strain from its plasmids.

Genus *Pantoea* was not so long ago separated from the genus *Enterobacter*, whose strains representing different species can be infected and lysogenized with P1 (Goldberg et al., 1974; Murooka and Harada, 1979; Tominaga and Enomoto, 1986; Bednarek et al., 2022). Moreover, circular sequences resembling the D6-like prophages, distantly related to P1, have been identified in a few *Pantoea* sp. strains (Gilcrease and Casjens, 2018, and references therein). Therefore, the inability of *P. agglomerans* L15 to be lysogenized with P1 *c1-100* Tn*9* was an unexpected feature of this strain. Additionally, the transfer of the Tn*9 cat* gene from the genome of P1 *c1-100* Tn*9* to the genome of L15 suggested that the mechanisms preventing the lysogeny act relatively late, leaving enough time for the interactions between the incoming DNA of P1 and the L15 chromosome. To identify these mechanisms we isolated the total DNA of *P. agglomerans* L15 and determined its complete genomic sequence using a hybrid approach (Illumina and MinIon; see Materials and methods). The assembly of sequence reads revealed four circular DNA molecules representing a chromosome (4,029,228 bp) and three plasmids of 583,567, 179,590, and 66,484 bp, that were designated by us as pPagL15_1, pPagL15_2, and pPagL15_3, respectively. The overall features of all determined sequences are summarized in Table 1 and Figure 2. The sequence of the L15 chromosome and the two largest plasmids appeared to be highly similar to the previously described chromosomal and plasmid sequences of other *P. agglomerans* strains isolated from different plants or soil (Table 1 and references therein). The sequence of the smallest plasmid was only up to 24% identical to the sequences of its most closely related plasmids from the GenBank database. The detailed description of the L15 chromosome and plasmidome coding potential, especially the identification of features that may be associated with the biocontrol properties of the L15 strain, will be described elsewhere. Here, we searched for genetic determinants of this strain and its plasmids that could be responsible for the inability of L15 to support the lysogeny by phage P1 *c1-100* Tn*9*.

**Table 1:** General features of the *P. agglomerans* L15 chromosome and plasmids.

**Genome part**	**Size (bp)**	**% GC**	**Number of predicted protein coding genes**	***rrn* operons (tRNA genes)**	**Closest relatives**	**Size (bp)**	**GenBank Acc. No. (reference)**	**% coverage/ % identity**
Chromosome	4,029,228	55.6	3,698	7 (75)	• *P. agglomerans* ASB05	4,022,781	CP046722 (Lee et al., 2021)	95/99
					*P. agglomerans* C410P1	4,182,028	CP016889	94/98
					*P. agglomerans* TH81	4,128,817	CP031649 (Humphrey et al., 2019)	94/99
pPagL15_1	583,567	53.0	614	0 (6)	• *P. agglomerans* C410P1 plasmid, unnamed1	543,504	CP016890	73/96
					*P. vegans* C9-1 plasmid pPag3	529,676	CP001895 (Smits et al., 2010)	63/87
pPagL15_2	179,590	53.0	163	0 (0)	• *P. agglomerans* ASB05 plasmid pASB05p2	207,454	CP046724 (Lee et al., 2021)	99/99
					• *P. agglomerans* PSV1-7, plasmid, unnamed2	179,440	CP091191	99/99
					• *P. agglomerans* CHTF15 plasmid, unnamed2	180,424	CP103403	99/99
					• *Pantoea vegans* C9-1 plasmid pPag1	167,983	CP001893 (Smits et al., 2010)	75/89
pPagL15_3	66,484	50.4	79	0 (0)	• *P. agglomerans* DAPP-PG734 plasmid P2	174,327	OW970317	22/96
					• *P. agglomerans ASB05* plasmid pASB05p2	64,606	CP046725 (Lee et al., 2021)	16/97
					*P. agglomerans* C410P1 plasmid, unnamed2	216,117	CP016891	26/91
					*Pantoea vegans* C9-1 plasmid pPag2	165,693c	CP001894 (Smits et al., 2010)	13/90
					*P. agglomerans* TH81 plasmid unnamed3	152,523	CP031652 (Humphrey et al., 2019)	18/92

Bacteria encode numerous anti-phage defense systems that act at the post-infection step to destroy the incoming phage DNA or to cause a suicide of the infected cell, preventing the infection from spreading. Our search revealed that the L15 chromosome encodes five predicted defense systems: Mokosh type II and Shango (Millman et al., 2022), RloC (Davidov and Kaufmann, 2008), and MazEF (Hazan and Engelberg-Kulka, 2004) and restriction-modification system of type I (Supplementary Table 5). Two other systems, the R-M system of type II and the Gao-ppl system (Gao et al., 2020), are encoded by the pPagL15_1 and pPagL15_3 plasmid, respectively. The Makosh, Shango, and Gao_ppl systems did not exhibit anti-phage activity against the P1 bacteriophage (Gao et al., 2020; Millman et al., 2022). RloC is an anticodon nuclease that targets the tRNA^Lys^ of the host cell to inhibit translation during the T4 infection (Klaiman et al., 2012). Yet, its influence on P1 infection has not been studied. The MazEF is a toxin-antitoxin system that significantly decreased the rate of P1 lysogen formation in *E. coli* (Hazan and Engelberg-Kulka, 2004). However, the action of the MazEF system caused the premature death of infected cells, but fewer stable lysogens could still be formed. A search for the CRISPR-Cas regions in the L15 chromosome and its plasmids revealed some positive incomplete matches. However, their predicted spacer regions did not have similarities to any P1-derived sequence fragment (data not shown).

Several genes of P1 that are present in lysogens encode proteins, preventing the superinfection of lysogens with P1 or related phages. To find possible homologs of such proteins, we searched predicted sequences of proteins encoded by the chromosome of L15 and its plasmids using BlastP and tBlastN programs and sequences of bacteriophage P1 proteins (Supplementary Table 6). Our search revealed that both the L15 chromosome and its plasmids encode proteins highly similar to the proteins of P1.

Chromosomally encoded homologs of P1 proteins include mainly proteins associated with replication. Homologs of these proteins are also encoded by the *E. coli* chromosome, and they support replication from the P1 plasmid or phage replication origin (Łobocka et al., 2004).

**Figure 3:**
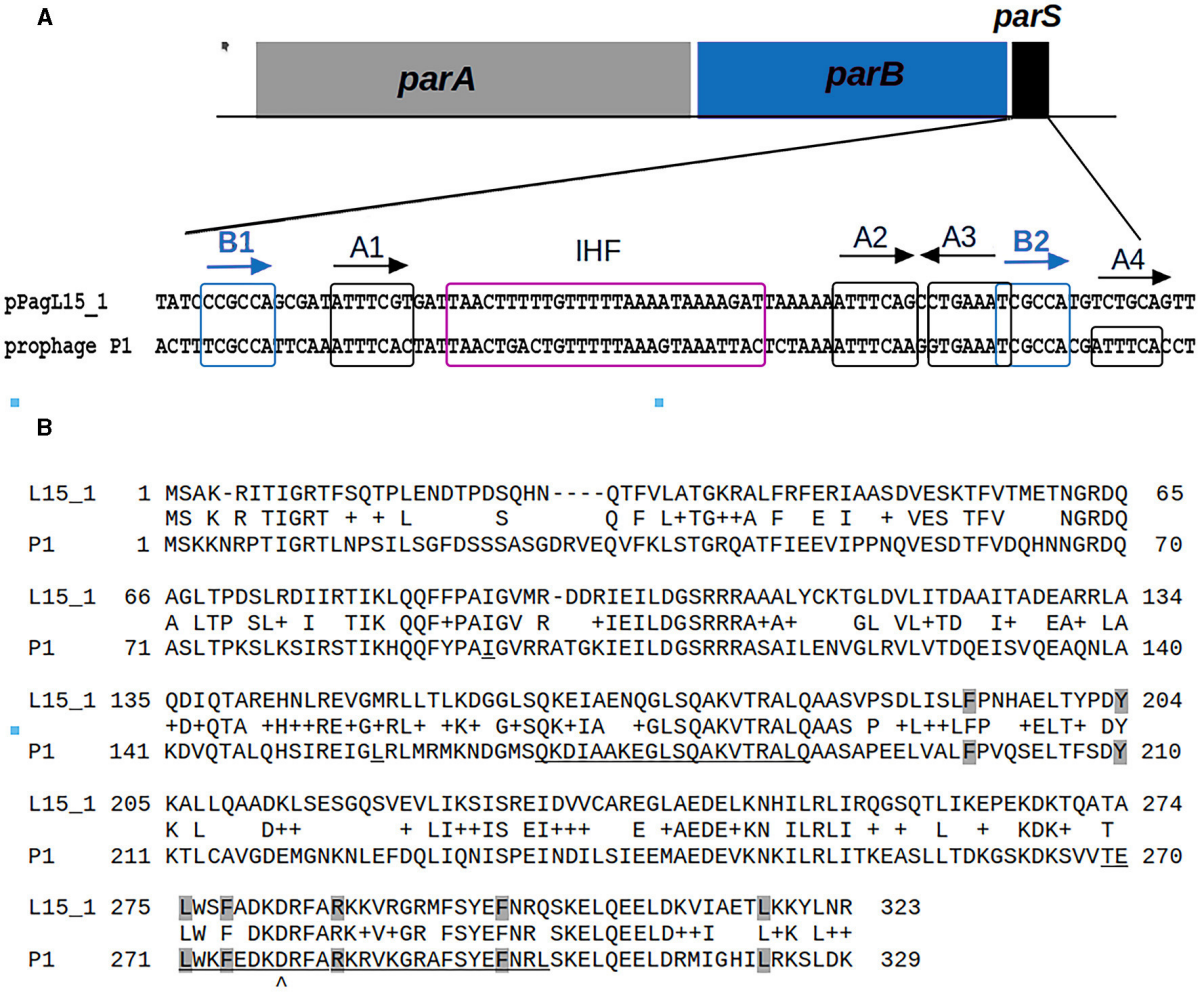
Similarities between the organization and sequence of the predicted partition cassette of pPagL15_1 plasmid and prophage P1. **(A)** Sequence alignment of the predicted centromere-like site of pPagL15_1 (*parSp*_PagL15_1_) and the *parS* site of P1. A scheme of partition cassettes is above the alignment. Boxes A and B which are involved in the binding of P1 ParB, and the predicted and known IHF binding sites of pPagL15_1 and P1, respectively, are indicated. Boxes B, essential for partition incompatibility determination, are boxed in blue. **(B)** Alignment of amino acid sequences of predicted ParB protein of pPagL15 and ParB protein of P1. Amino acid residues of P1 ParB shown by mutational replacements as essential for binding with *parS* and their counterparts in pPagL15_1 ParB are shaded in gray. Regions of P1 ParB essential for the specific recognition of P1 *parS* are underlined. The aspartic acid residue of the P1 ParB, key for recognizing the second base of species-specificity determining BoxB2, is indicated below the alignment. The essential partition specificity and incompatibility determining regions of P1 ParB are indicated according to Radnedge et al. (1998) and Dabrazhynetskaya et al. (2009).

Surprisingly, two plasmids of the L15 strain appeared to encode homologs of P1 proteins that could potentially contribute to interactions with P1 responsible for the difficulties in establishing P1 lysogeny. The pPagL15_1 plasmid encodes proteins highly similar to the partition proteins of P1, ParA, and ParB (Supplementary Table 6, Figure 3). In the genome of P1, immediately downstream of the *parB* gene, there is a ParB binding site, *parS*, which acts as an analog of the eukaryotic centromere (Abeles et al., 1985). The binding of ParB to *par*S is essential for active plasmid segregation. Immediately downstream of the *parB* gene of pPagL15_1, there is a sequence nearly identical to the *parS* site of prophage P1 (designated here as *parS*_pPagL15_1_; Figure 3A). In P1 *parS* there are two ParB binding sites—heptameric sites designated as BoxA and hexameric sites designated as BoxB. They are organized in two clusters (BA and AABA) and separated by the site that binds a host factor, IHF protein. Only A2-A3 and B2 boxes (forming the so-called *parS* small) are required to bind ParB. The BoxB sequences are discriminatory elements that are responsible for the specific interaction of ParB with the *parS* site of P1 but not with the related *parS* sequences of other plasmids (Dabrazhynetskaya et al., 2005, 2009; Sergueev et al., 2005). The *parS*_pPagL15_1_ site appears to be organized similarly. It contains all A and B boxes shown to be essential for P1 partitioning and the specific binding of P1 ParB. Box B2, which was shown to be the only B box essential for determining the P1 specific partition incompatibility (Dabrazhynetskaya et al., 2005), is identical in P1 *parS* and pPagL15_1 *parS*. Moreover, the amino acid residues of P1 ParB, which are essential for binding to *parS* and are involved in discriminating *parS* of P1 from the related *parS* sites of other plasmids (Łobocka and Yarmolinsky, 1996; Radnedge et al., 1998; Dabrazhynetskaya et al., 2005, 2009), are conserved in the ParB of pPagL15_1 (Figure 3B). Taken together this indicates that prophage P1 and plasmid pPagL15_1 are partitionally incompatible and cannot stably coexist in one cell.

**Figure 4:**
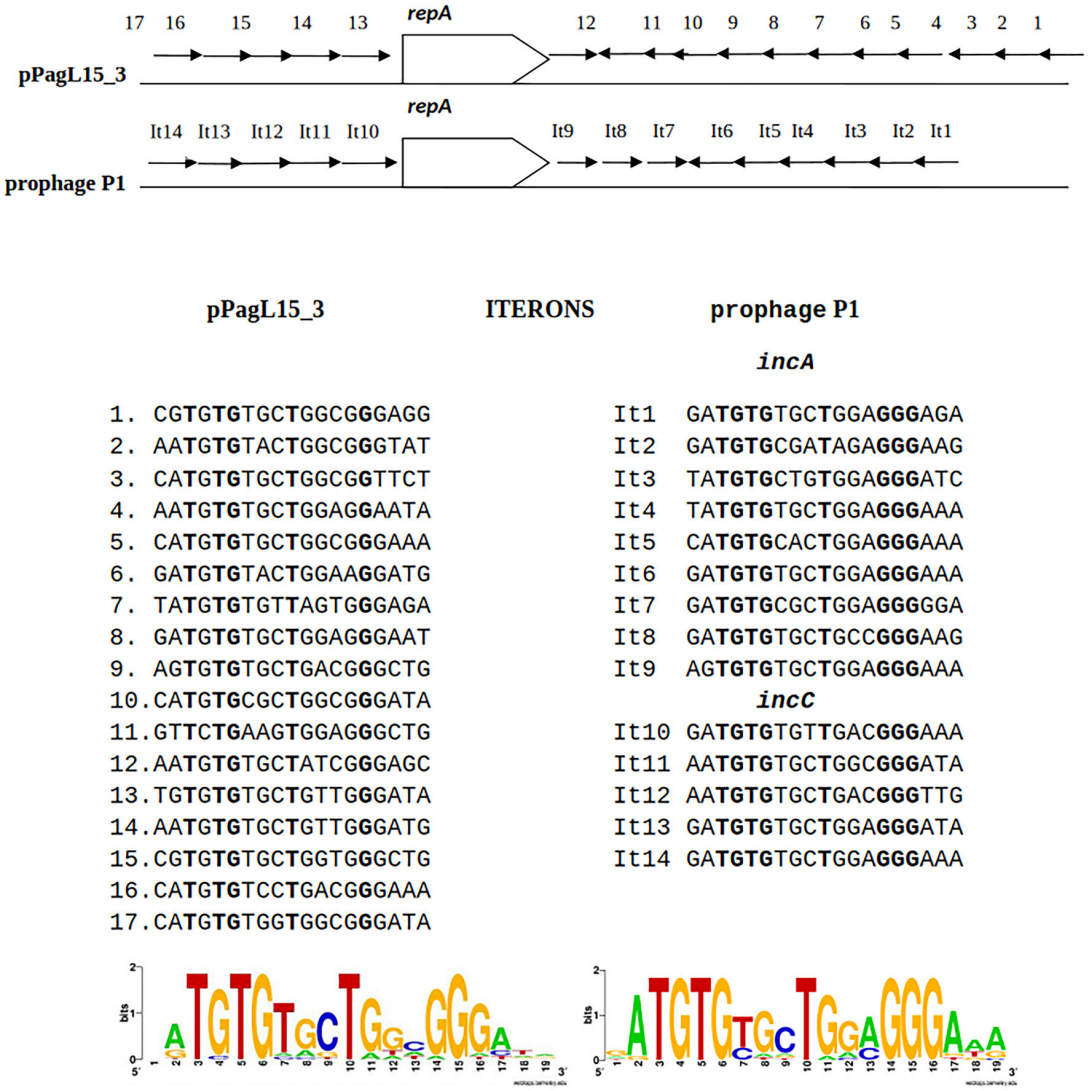
Similarities between the organization of the predicted replication initiation region of plasmid pPagL15_3 and the replication initiation region of plasmid-prophage P1. Arrows in the regions flanking the *repA* gene indicate the organization and direction of iteron sequences. Sequences of iterons of pPagL15_3 and prophage P1 and their logos are below the comparative scheme.

The predicted replication initiator protein of pPagL15_3 plasmid, designated here as RepA_pPagL15_3_, is 73% identical over 97% of its length to the RepA replication initiator protein of phage P1 (Figure 4). Additionally, the so-called iteron sequences that, by prediction, bind this protein by analogy to other plasmids replicating in the theta mode, are nearly identical to the iteron sequences binding the RepA protein of P1 (Figure 4). The aforementioned similarities indicate that the pPagL15_3 plasmid and prophage P1 belong to the same replication incompatibility group (IncY) and cannot be stably maintained in the same cell. Certain other RepA proteins of IncY group plasmids of experimentally proven incompatibility with P1 are also identical 72–73% to P1 RepA at the protein sequence level and recognize iterons similar to those of P1 (Fu et al., 1997).

### 3.5 Curing of *P. agglomerans* L15 strain from the pPagL15_1 plasmid which is partitionally incompatible with prophage P1

**Figure 5:**
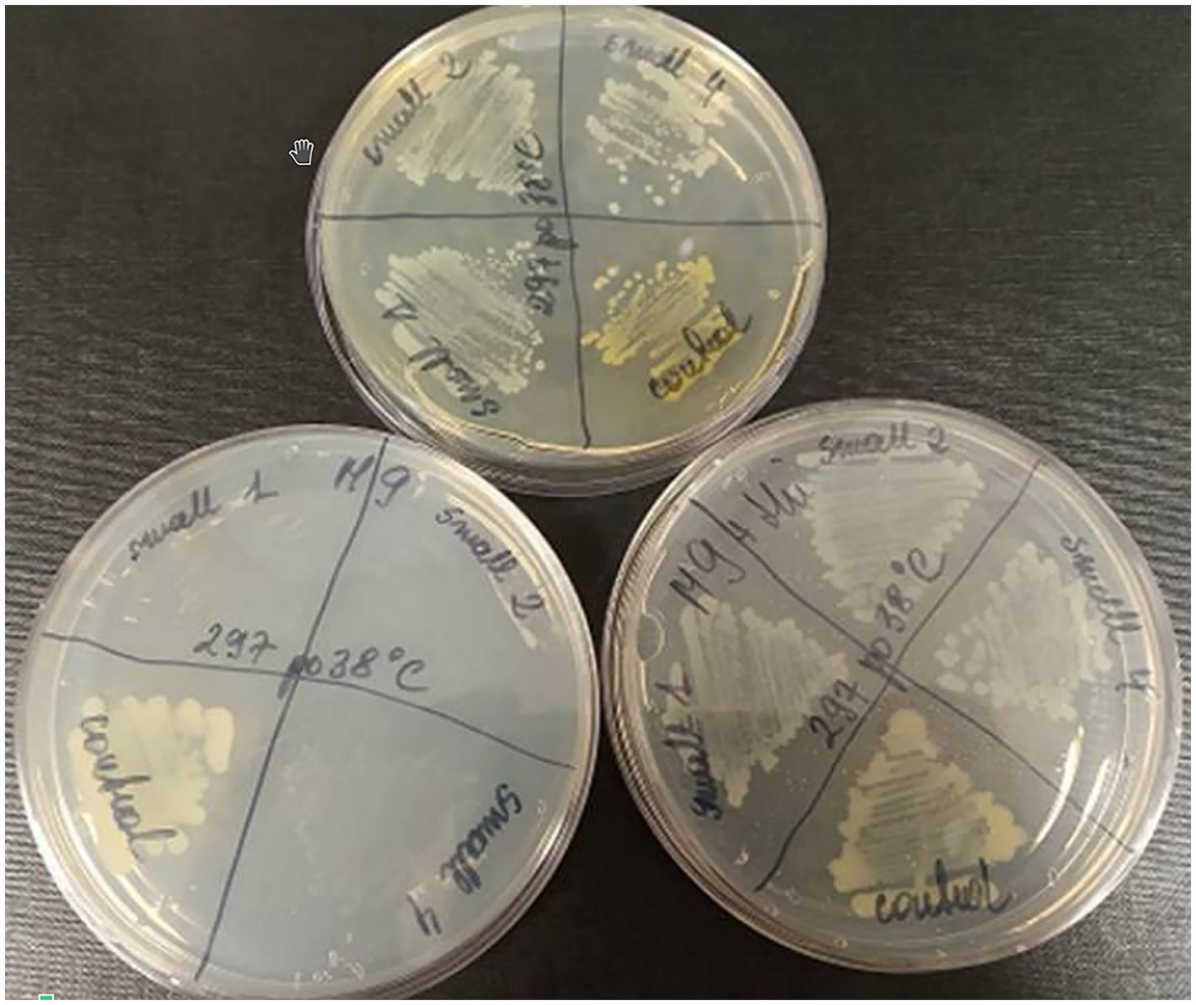
Curing of *P. agglomerans* L15 strain from the pPagL15_1 plasmid. Curing was based on screening for colonies that lost the ability to produce yellow stain (zeaxanthin) and to synthesize thiamine encoded by *crtX* and *thiOSGF* genes of pPagL15_1, respectively. The phenotypes of pPagL15_1-cured clones of L15 that were obtained after incubation of *P. agglomerans* L15 at 35°C for about 22 generations, plated on LB medium to obtain single colonies and screened for the presence of white clones. **(Upper plate)** shows differences in the colony color of pPagL15_1-cured clones and the parental L15 strain. Two plates at the bottom show the inability of pPagL15_1-cured clones to grow on a minimal M9 medium supplemented with glucose but without thiamine **(left plate)** and their ability to grow on a similar medium supplemented with thiamine **(right plate)**.

To test whether the presence of the L15 strain of the two plasmids incompatible with prophage P1 could be responsible for the inability of P1 to stably lysogenize this strain, we first attempted to cure the L15 strain from the pPagL15_1 plasmid, which was partitionally incompatible with P1. This plasmid encodes at least two phenotypic markers, namely the synthesis pathway of pigment zeaxanthin, which is responsible for the yellow coloring of the L15 colonies, and the thiamine biosynthesis pathway (Figure 2). Thus, to cure the L15 cells from this plasmid, we incubated them at 35°C (the highest temperature tolerated by L15) and plated them on an LA medium to obtain single colonies. Of over 9,000 single colonies inspected, six did not show yellow coloring. Additionally, when tested for the ability to grow on a minimal solid M9 medium with glucose and with or without thiamine, they could only grow on the medium with thiamine, contrary to the wild-type L15 cells that could grow on both these media (Figure 5). Shotgun WGS sequencing of the DNA of one of the obtained strains confirmed the absence of DNA fragments representing the pPagL15_1 plasmid in its cells (Supplementary Table 7). We designated the obtained L15 derivative cured of pPagL15_1 plasmid as IPAG312.

Our attempts to obtain stable P1 *c1-100* Tn*9* lysogens of the IPAG312 strain appeared unsuccessful. As in previous attempts, we could obtain colonies grown on LA medium with chloramphenicol. However, testing them for the presence of P1 *c1-100* Tn*9* and the pPagL15_3 plasmid after several generations of growth in chloramphenicol-containing medium by colony PCR with primers specific for P1 *c1-100* Tn*9* genes and pPagL15_3 genes revealed that they contained only the *cat* gene of P1, and retained the pPagL15_3 plasmid.

### 3.6 P1 *c1-100* Tn*9* derivatives able to outcompete plasmid pPagL15_3 from the *P. agglomerans* IPAG312 cells

Replication or partition incompatibility leads to a random loss of any of the incompatible plasmids of a pair at cell divisions (Novick, 1987). The inability of P1 *c1-100* Tn*9* to replace the pPagL15_3 plasmid in the IPAG312 cells could suggest a mechanism protecting pPagL15_3 from the loss. We excluded that pPagL15_3 could be a mini-chromosome. Homologs of all *E. coli* K-12 substr. MG1655 proteins deposited in the database of essential *E. coli* gene products (https://shigen.nig.ac.jp/ecoli/pec/) could be identified among the predicted gene products of *P. agglomerans* L15 chromosome.

The pPagL15_3 plasmid encodes two proteins significantly similar to proteins of the canonical CcdACcdB type II TA system of F plasmid. The pPagL15_3 homolog of CcdA (WP_033781302.1; 78 aa) is 28% identical to F plasmid CcdA (WP_000813634.1; 72 aa), and the pPagL15_3 homolog of CcdB (WP_031594618.1; 104 aa) is 37% identical to the F plasmid CcdB (WP_031594618.1; 101 aa). Additionally, the amino acid sequences of pPagL15_3 CcdA and CcdB homologs contain amino acid sequence motifs characteristic of CcdA and CcdB family proteins, pfam motif PF07362 and PF01845, respectively. CcdB is a potent toxin that eliminates cells with plasmids that express its gene and do not contain the CcdA antitoxin (Bernard, 1996). To determine whether the inability of P1 *c1-100* Tn*9* to outcompete the pPagL15_3 plasmid from the IPAG312 cells could be due to the CcdB-mediated killing of cells that potentially lost the pPagL15_3, we constructed a derivative of P1 *c1-100* Tn*9* with the pPagL15_3 plasmid *ccdA* gene with its promoter inserted in the IS*1*L sequence of Tn*9*. When the IPAG312 cells were infected with the P1 *c1-100* Tn*9* IS*1*::*ccdA*_pPagL15_3_ bacteriophage, incubated to express the *cat* gene of Tn*9* and plated on medium with chloramphenicol, chloramphenicol-resistant colonies showed up after overnight incubation similar to the experiments with P1 *c1-100* Tn*9*. However, when we purified these colonies on a similar medium and used them to inoculate the LB medium with chloramphenicol, all of them grew to a stationary phase without noticeable growth retardation. To test whether the obtained clones could lose the pPagL15_3 plasmid after prolonged incubation under conditions selective for the P1 *c1-100* Tn*9* IS*1*::*ccdA*_pPagL15_3_ prophage, we restreaked them to M9 minimal medium supplemented with glucose, thiamine, and chloramphenicol, and then again on LA medium with chloramphenicol to obtain single colonies. Cells from the top of these colonies were used as a source of template DNA in colony PCR reactions with primers specific for P1 and pPagL15_3 DNA. In all tested cases, the amplicons were formed only with primers specific for P1, indicating that all tested clones acquired the P1 *c1-100* Tn*9* IS*1*::*ccdA*_pPagL15_3_ prophage and lost the pPagL15_3 plasmid (Supplementary Figure 7).

To verify whether the ability of P1 *c1-100* Tn*9* IS*1*::*ccdA*_pPagL15_3_ to replace the pPagL15_3 plasmid in the obtained clones resulted from the presence of the source of CcdA antitoxin in the modified P1 *c1-100* Tn*9* construct used for curing, we constructed another P1 *c1-100* Tn*9* bacteriophage derivative with the kanamycin resistance cassette inserted in the IS*1*L sequence of Tn*9*. Surprisingly, the results were similar to those obtained with P1 *c1-100* Tn*9* IS*1*::*ccdA*_pPagL15_3_. The IPAG312 strain cells lysogenized with P1 *c1-100* Tn*9* IS*1*::km^R^ formed colonies on the LA medium with chloramphenicol and kanamycin. When cells of these colonies were used to inoculate LB medium with chloramphenicol and kanamycin, the optical density of the obtained cultures increased as expected. We attempted to cure them of pPagL15_3 plasmid by restreaking them on M9 solid medium with glucose, thiamine, kanamycin, and chloramphenicol and restreaking again on LA medium with chloramphenicol and kanamycin. When cells from colonies grown on LA plates with chloramphenicol and kanamycin were used as a source of template DNA in colony PCR with primers specific for P1 and for the PagL15_3 plasmid, the expected amplicons were obtained only with primers specific for P1, indicating that they lost the pPagL15_3 plasmid. One of the obtained lysogens cured of pPagL15_3 and containing P1 *c1-100* Tn*9* IS*1*::km^R^ prophage (designated by us as IPAG380) was used as a source of template DNA for long-read diagnostic shotgun WGS sequencing. Analysis of sequencing results confirmed that it represents a derivative of *P. agglomerans* IPAG312 strain cured of pPagL15_1 and pPagL15_3 plasmids and lysogenized with P1 *c1-100* Tn*9* IS*1*::km^R^ (Supplementary Table 7). Clearly, the reason for P1 *c1-100* Tn*9* being unable to stably lysogenize the IPAG312 strain was the IS*1*L sequence of the P1 mutant Tn*9*.

The IS1*L* sequence of Tn9 proved to be the only Tn*9* IS*1* sequence driving the mobility of Tn*9* (Chandler and Galas, 1983; Machida et al., 1983; Ahmed, 1984). Therefore, to determine whether IS*1*L by itself or the Tn*9* mobility driven by IS*1*L is the reason for the P1 *c1-100* Tn*9* inability to stably lysogenize *P. agglomerans* L15 or IPAG312, we infected the IPAG312 cells with a derivative of P1 *c1-100* Tn*9* containing the insertion of kanamycin resistance cassette in the non-essential *pdcB* gene and constructed by us previously (Bednarek et al., 2023). The infected cells could form colonies on the LA medium with kanamycin or with chloramphenicol and kanamycin. Moreover, when these colonies were used to inoculate liquid medium with kanamycin or chloramphenicol and kanamycin, their culture grew without any retardation, indicating that they formed stable lysogens. Consequently, our results show that the reason for P1 *c1-100* Tn*9* inability to stably lysogenize *P. agglomerans* IPAG312 cells is the mobility of its selective marker, namely the Tn*9 cat* gene. The immobilization of Tn*9* or the enrichment of P1 *c1-100* Tn*9* with an immobile selective marker makes P1 a winner in the fight with incompatible plasmids to settle the L15 cells as the hosts.

### 3.7 The ability of P1 *c1-100* Tn*9* IS*1*::km^*R*^ to replace two incompatible plasmids and to develop lytically in the *P. agglomerans* L15 cells

To test whether the P1 *c1-100* Tn*9* IS*1*::km^R^ bacteriophage can stably lysogenize the *P. agglomerans* L15 strain and replace both plasmids of this strain incompatible with P1, we repeated the lysogenization procedure with this phage using the L15 strain as a recipient in the lysogenization. Similarly, as in the case of the IPAG312 strain, stable lysogens of L15 with P1 *c1-100* Tn*9* IS*1*::km^R^ could be obtained, and they could grow on LA medium with chloramphenicol and kanamycin as well as in liquid medium supplemented with these antibiotics. Moreover, after a few rounds of their streak plating on LA medium with chloramphenicol and kanamycin, they gave rise to colonies of cells cured of both pPagL15_1 and pPagL15_3 plasmids, as verified by PCR and by shotgun WGS sequencing (Supplementary Figure 7, Supplementary Table 7).

**Figure 6:**
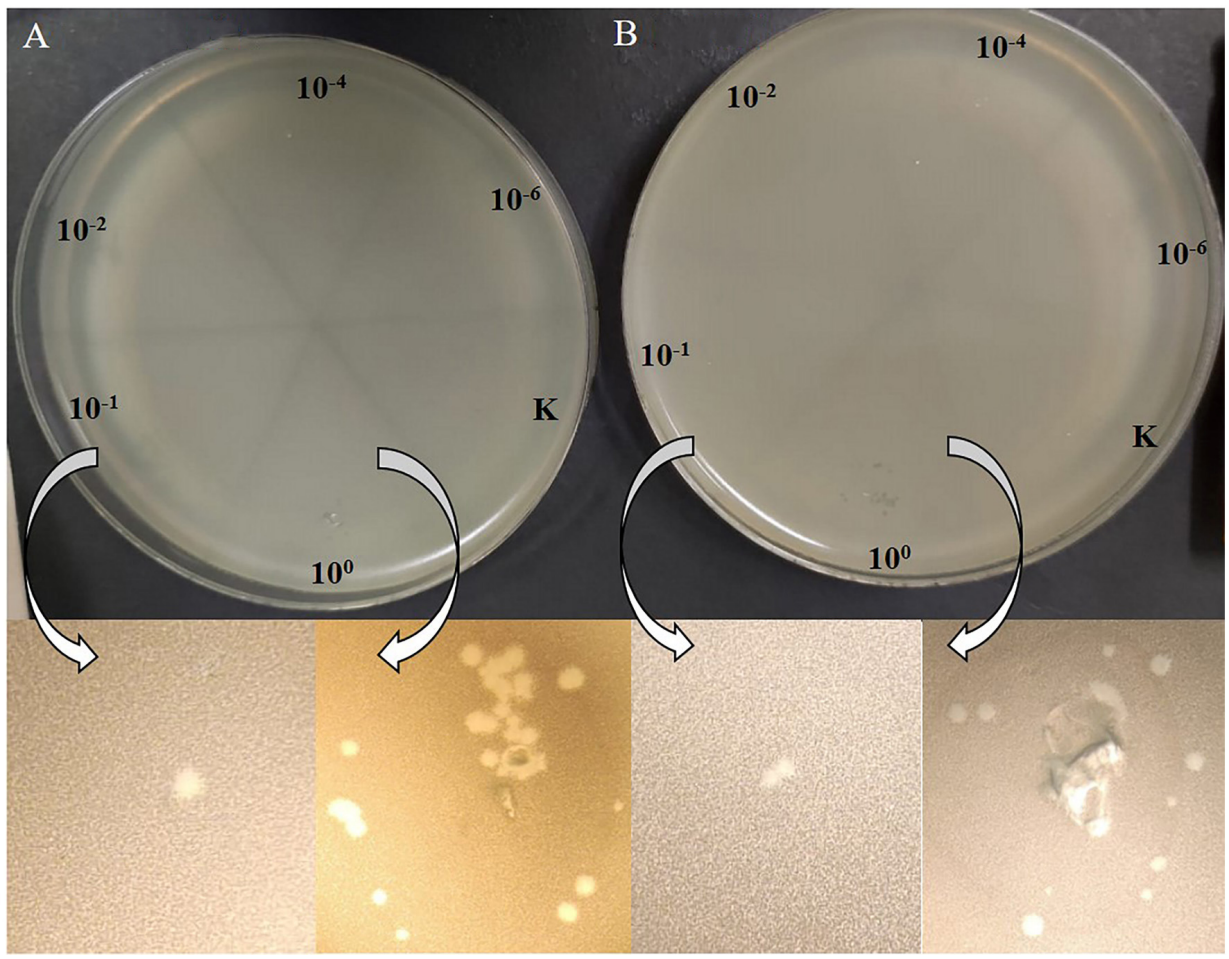
Plaques formed by P1 *c1-100* Tn*9* IS*1*::km^R^ bacteriophage released from *P. agglomerans* L15 and *P. agglomerans* IPAG312 lysogens on the layer of *E. coli* N99 indicator strain cells. **(A)** IPAG312/P1. **(B)** L15/P1.

The acquisition of *P. agglomerans* L15 and IPAG312 strain derivatives cured of plasmids incompatible with P1 and lysogenized with P1 *c1-100* Tn*9* IS*1*::km^R^ prompted us to test whether P1 can develop lytically in the cells of these strains. Cultures of lysogens in liquid LB medium with chloramphenicol and kanamycin were grown with shaking until their optical density (OD_600_) reached ~0.5. They were then quickly heated to 43°C to induce the prophage, incubated at 42°C with shaking for an additional 15 min, transferred to 33°C, and incubated for an additional 70 min with shaking. The aliquots of the cultures were serially diluted and spotted on a layer of indicator strain cells (*E. coli* N99) in LCA in Petri dishes. The plates were incubated overnight at 42°C and inspected for plaques. Surprisingly, in both cases, single plaques were obtained in contrast to the control samples in which we used the aliquots of the cultures of non-lysogens for spotting (Figure 6). To sum up, our results demonstrate that bacteriophage P1 can not only stably lysogenize *P. agglomerans* L15 cells but can also develop lytically in the cells of this strain. Additionally, the ability of P1 *c1-100* Tn*9* IS*1*::km^R^ to replace the pPagL15_1 and pPagL15_3 plasmids in the L15 strain confirms our predictions that each of these plasmid is incompatible with prophage P1.

### 3.8 Nucleotide sequence accession number

The genomic sequences of the *P. agglomerans* L15 chromosome, and the pPagL15_1, pPagL15_2, and pPagL15_3 plasmids were deposited in GenBank under the accession numbers CP034148, CP034149, CP034150, and CP034151, respectively.

## 4 Discussion

In this work, we showed that an epiphytic isolate of *P. agglomerans*—namely, the *P. agglomerans* L15 strain can be added to the list of bacteriophage P1 hosts. We also demonstrated that P1 can be a vector for transferring plasmids from a laboratory *E. coli* strain to the *P. agglomerans* L15 strain. Additionally, the determination of the complete *P. agglomerans* L15 genomic sequence, and the analysis of the L15 strain interaction with the P1 *c1-100* Tn*9* mutant phage allowed us to discover an unexpected influence of antibiotic selection pressure on the interplay between the incoming and resident mobile genetic elements and on breaking natural barriers protecting cells from the establishment of foreign DNA.

In *E. coli* K-12, P1 adsorbs to the terminal glucose in the outer core oligosaccharide of LPS (Franklin, 1969; Ornellas and Stocker, 1974; Yarmolinsky and Sternberg, 1988, and references therein). The core oligosaccharide of *P. agglomerans* LPS is highly diversified. In the case of several strains described, it is similar to that of *E. coli* in that it contains glucose in the core oligosaccharide (Karamanos et al., 1992; Lerouge and Vanderleyden, 2002; Kohchi et al., 2006; Hashimoto et al., 2017; Varbanets et al., 2019; Bulyhina et al., 2020). Therefore, it was unsurprising that P1 adsorption to *P. agglomerans* L15 cells was possible and similar to that of *E. coli* K-12 cells concerning time and efficiency (Supplementary Figure 1). Likely, not all *P. agglomerans* strains can adsorb P1, as not all contain glucose in their core LPS oligosaccharide (Varbanets et al., 2019). However, P1 requires only one receptor-binding protein (tail fiber) to adsorb to a host cell and inject its DNA (Liu et al., 2011; Lam et al., 2021). This offers a relatively simple system for phage specificity engineering by modifying tail fiber modules essential for the direct interaction of tail fibers with host receptors for a phage, as has been demonstrated recently (Lam et al., 2021). Additionally, two alternative forms of the C-terminal fragment of tail fiber protein, naturally encoded by the invertible C-segment of P1, enable the expression of two-tail fiber variants by P1 progeny (reviewed by Łobocka et al., 2004). Whether the alternative P1 tail-fiber variant may be required for the adsorption to *P. agglomerans* strains of atypical LPS core oligosaccharide structure remains to be found.

Despite the ability of P1 to adsorb to the L15 cells and the close phylogenetic relationship of *P. agglomerans* and *E. coli* (the best-known host of P1), we could not obtain stable L15 lysogens when we lysogenized L15 using the thermo-inducible P1 mutant, P1 *c1-100* Tn*9*, which carries the chloramphenicol resistance marker in Tn*9*. All obtained clones that became stably resistant to chloramphenicol with time carried only the chromosomally integrated and shortened versions of Tn*9*. It contained the chloramphenicol resistance marker but was depleted of the leftmost IS*1* sequence. Of the two IS*1* sequences at the ends of the Tn*9* transposon, the leftmost one (IS*1L*), preceding the *cat* gene promoter, is active, while the rightmost one (IS*1R)* is not, due to the transcriptional repression from the *cat* gene promoter (Chandler and Galas, 1983; Machida et al., 1983; Ahmed, 1984). We suppose that due to the presence in the L15 cells of two plasmids incompatible with P1 and the use of chloramphenicol to select lysogens, only cells in which Tn*9* transposed to the chromosome of L15 strain before the donor P1 loss could survive in the selective condition for a longer time. This could also be a reason why, despite similar adsorption efficiency of bacteriophage P1 *c1-100* Tn*9* to *E. coli* and *P. agglomerans* L15 cells, the number of colonies grown on selective medium after infection of *E. coli* was one order of magnitude higher than that grown after infection of *P. agglomerans* L15.

The truncated Tn*9* insertions in the L15 chromosome, in nine independent clones, were mapped to various sites of the region between pos. 3552017–3552106 of the *P. agglomerans* L15 genome corresponding to the promoter region and the 5′ end of a gene encoding the YdgH/BhsA/McbA-like domain-containing protein of unknown function (locus_tag CBF16_RS16745; Figure 1). The preferential insertion of Tn*9* in one locus is consistent with previous observations that while in general IS*1* and Tn*9* insert into easily denaturable regions with some specificity to the sequences homologous to the inverted terminal repeats (ITRs) of these elements (Galas et al., 1980; Miller et al., 1980), in some strains they insert preferentially in one locus (Il'ina et al., 1980). We found that the 7-nt sequence (TGCCAAC) present in the leftmost ITRs of both IS*1* sequences of P1 Tn*9* is also present at pos. 3552102 of the *P. agglomerans* L15 genome and marks the border between the L15-derived and Tn9-derived sequence in two of the analyzed clones. However, this sequence is present in 410 differently localized and differently oriented copies in the L15 chromosome, suggesting that its presence in the insertion region is either unimportant or is not the only factor responsible for selecting this insertion site.

The insertions of Tn*9* from P1 to the L15 chromosome studied in this work were always associated with deletions of the leftmost IS*1* sequence of Tn*9* and various parts of the Tn*9* region preceding the *cat* gene promoter. Additionally, they were mostly accompanied by the rearrangement in the remaining parts of this region in Tn*9*. This is consistent with previous observations indicating that the Tn*9* as well as IS*1* insertions cause alterations in the insertion target sites (Johnsrud et al., 1978; Ohtsubo and Ohtsubo, 1978; Calos and Miller, 1980; Meyer et al., 1980; Iida et al., 1981; Résibois et al., 1981; Gorb et al., 2011). The Tn*9* insertion in the P1 *c1-100* Tn*9* bacteriophage, which was used in this study, is at the resident IS*1* of P1 and contains the P1-derived IS*1* sequence at its left border (Galas et al., 1980; Łobocka et al., 2004). It was formed not by Tn*9* transposition to the genome of P1 but by two successive recombinations between the P1 IS*1* and the IS*1* sequences of R plasmid pSM14, which served as a donor of chloramphenicol resistance marker to P1 (Iida, 1983). Possibly, the P1 Tn*9* insertions in a single region of the *P. agglomerans* L15 strain are also formed by recombination.

We found that the epiphytic environmental *P. agglomerans* L15 strain can become proficient for the lysogeny with P1 *c1-100* Tn*9* phage if obstacles preventing the lysogeny can be overcome. The only obstacle preventing the lysogeny appears to be the mobility of the P1 Tn*9* transposon. One can assume that conditions promoting the transposition of Tn*9* to a chromosome remove the selective pressure to maintain the P1 prophage in the freshly formed lysogens. Additionally, the accompanying deletion of the Tn*9* leftmost IS*1*, which is responsible for transposon mobility, prevents the reinsertion of truncated Tn*9* into the P1 genome. Together, this leads to the out-competition of P1 by the resident plasmids that are incompatible with it. We show that the immobilization of P1 Tn9 or the selection for a different immobile antibiotic-resistant marker inserted in the P1 *c1-100* Tn*9* genome, in addition to Tn*9*, reverts this scenario by enabling infecting P1 to outcompete both plasmids incompatible with it and to establish lysogeny. The antibiotic-dependent selective pressure to keep P1 in cells has a much stronger effect on the outcome of P1 infection than the presence in a cell of MazEF anti-phage defense system and of plasmids incompatible with P1. This may explain, at least in part, a surprisingly fast spreading of antibiotic resistance in bacteria grown in environments contaminated with antibiotics.

The P1 *c1-100* Tn*9* mutant has been widely used in studies on the host range of P1 (Yarmolinsky and Sternberg, 1988; Giermasińska and Łobocka, 2016, and references therein). Here, we show that even if bacteria can adsorb P1 and are metabolically compatible with P1 to support its lytic development or lysogeny, this mutant's failure to lysogenize them may result from the action of factors selective to cells in which Tn*9* transposed to a chromosome. Thus, negative results of attempts to obtain P1 *c1-100* Tn*9* lysogens should be verified using a P1 *c1-100* derivative carrying an immobile antibiotic resistance marker.

An unexpected result of our study was the discovery of how easily P1 could outcompete the two plasmids incompatible with it from the *P. agglomerans* L15 cells if only it contained an immobile selective marker. This observation could find practical use in the future. Large plasmids are common in *Pantoea agglomerans* and other species of *Pantoea* genus (Shetty et al., 2023). Only a few of them have been studied and found to significantly contribute to the versatility of *Pantoea* spp. strains. Our analysis of *Pantoea* strain sequences deposited in GenBank (accessed 2023-12-06) revealed that plasmids with partition cassette or replicon sequences highly similar to those of pPagL15_1 and pPagL15_3, respectively, are common in *Pantoea* strains representing various species. Eighty-eight *Pantoea* plasmids encode proteins with 72–100% identity over their entire length to ParB of pPagL15_1 plasmid (Supplementary Table 8). In all these proteins, amino acid residues of P1 ParB conserved in pPagL15_1 ParB and shown in P1 ParB to be essential for binding to *parS* and for the determination of P1 specific incompatibility are conserved (data not shown). It is worth noting that all the *Pantoea* P1 ParB homologs are encoded by *Pantoea* megaplasmids (Supplementary Table 8). Plasmids with replicon sequences highly similar to pPagL15_3 (100% coverage and over 90% of identity) and likely to be replicationally incompatible with P1 are less common in *Pantoea* strains. We detected such sequences in 19 *Pantoea* plasmid genomes deposited in GenBank (accessed November 13, 2023). Seventeen of them were detected in *P. agglomerans*, one in *Pantoea vagans*, and one in *Pantoea alfalfa* (data not shown). Curing bacteria from plasmids lacking phenotypic markers that are easy to select or differentiate in simple plate tests is typically a challenge, especially if their stability is high. Thus, the P1 *c1-100* Tn*9* IS*1*::km^R^ mutant with immobilized Tn*9* may be a ready-to-use tool in curing *Pantoea* strains from plasmids incompatible with P1 to facilitate their functional analysis.

Our results indicate that P1 can enrich the repertoire of molecular biology tools available for genetic manipulations in *P. agglomerans*. Certain plasmid-based tools have been described as suitable for such manipulations. Some, but not all, *P. agglomerans* strains can stably maintain *E. coli* pBR322 plasmid-based vectors and can be electroporated with derivatives of pBR322 (Wright et al., 2001). Some others can be electroporated with shuttle vectors constructed by cloning in the *E. coli* pUC18 plasmid of small cryptic plasmids identified in certain endophytic *P. agglomerans* strains (Andreote et al., 2008; de Lima Procópio et al., 2011). However, the use of the tools mentioned above is limited due to the limit of cloned insert size and the failure of their introduction to certain *P. agglomerans* strains.

Meanwhile, we demonstrated here using about 12 kb plasmid, just as an example, that P1 phages can transfer plasmids to some *P. agglomerans* strains by transduction. In this study, we used homologous recombination to insert a plasmid into P1 DNA without compromising the phage function of P1, which limited the size of the insert. However, P1 is a phage that mediates high-efficiency generalized transduction (Kenzaka et al., 2007; Thomason et al., 2007 and references therein). Its head can accommodate DNA of ~105 kb and can pack large plasmids or chromosomal fragments of up to this size if they contain sequences resembling the P1 *pac* site to initiate packaging (Sternberg and Maurer, 1991; Coren et al., 1995; Huang and Masters, 2014). Thus, our results showing the ability of P1 with the stable, selective marker to infect the *P. agglomerans* L15 cells productively open a possibility to use P1 to transfer genomic fragments or plasmids between various *P. agglomerans* strains and between *P. agglomerans* and other hosts of P1, especially model *E. coli* strains. The only described *P. agglomerans* phage that mediates generalized transduction is a flagellotrophic phage that could not infect a tested *E. coli* strain (Evans et al., 2010). The capacity of the P1 head makes this phage potentially proficient to transfer from *P. agglomerans* to *E. coli*, even large gene clusters, such as those that encode antibiotic synthesis pathways that have been identified in certain *P. agglomerans* strains (see Williams et al., 2020, and references therein). Comparison of the *P. agglomerans* L15 chromosomal sequence with the genomic sequence of the *E. coli* K-12 MG1655 strain (NC_000913) using BlastN revealed 775 fragments that encompass 22% of the *P. agglomerans* L15 chromosome and are ~91% identical to those of *E. coli*, including several fragments of 100% identity. Thus, the P1-mediated transfer of certain selectable *P. agglomerans* genomic fragments to *E. coli* could be possible.

## Data availability statement

The datasets presented in this study can be found in online repositories. The names of the repository/repositories and accession number(s) can be found at: https://www.ncbi.nlm.nih.gov/genbank/, CP034148, CP034149, CP034150, and CP034151.

## Author contributions

KG-B: Conceptualization, Formal analysis, Funding acquisition, Investigation, Methodology, Validation, Visualization, Writing – original draft, Writing – review & editing, Data curation. JG: Data curation, Investigation, Methodology, Validation, Writing – original draft, Resources, Software. ES: Investigation, Methodology, Software, Writing – original draft, Data curation. UG: Investigation, Writing – original draft. KŻ: Investigation, Writing – original draft. HR-B: Resources, Writing – original draft. RG: Resources, Methodology, Writing – original draft. MŁ: Methodology, Resources, Conceptualization, Formal analysis, Funding acquisition, Investigation, Project administration, Software, Supervision, Validation, Visualization, Writing – original draft, Writing – review & editing, Data curation.

## References

[B1] Abby S. S. Néron B. Ménager H. Touchon M. Rocha E. P. (2014). MacSyFinder: a program to mine genomes for molecular systems with an application to CRISPR-Cas systems. PLoS ONE 9:e110726. 10.1371/journal.pone.0110726 25330359 PMC4201578

[B2] Abeles A. L. Friedman S. A. Austin S. J. (1985). Partition of unit-copy miniplasmids to daughter cells. III. The DNA sequence and functional organization of the P1 partition region. J. Mol. Biol. 185, 261–272. 10.1016/0022-2836(85)90402-4 3903163

[B3] Adams M. H. (1959). Bacteriophages. New York, NY: Interscience Publishers Inc.

[B4] Ahmed A. (1984). A deletion analysis of transposon Tn*9*. J. Mol. Biol. 173, 523–529. 10.1016/0022-2836(84)90395-4 6323721

[B5] Altschul S. F. Madden T. L. Schäffer A. A. Zhang J. Zhang Z. Miller W.. (1997). Gapped BLAST and PSI-BLAST: a new generation of protein database search programs. Nucleic Acids Res. 25, 3389–3402. 10.1093/nar/25.17.3389 9254694 PMC146917

[B6] Andreote F. D. Rossetto P. B. Souza L. C. Marcon J. Maccheroni W. Jr. Azevedo J. L.. (2008). Endophytic population of Pantoea agglomerans in citrus plants and development of a cloning vector for endophytes. J. Basic Microbiol. 48, 338–346. 10.1002/jobm.200700341 18759238

[B7] Aziz R. K. Bartels D. Best A. A. DeJongh M. Disz T. Edwards R. A.. (2008). The RAST Server: rapid annotations using subsystems technology. BMC Genom. 9:75. 10.1186/1471-2164-9-75 18261238 PMC2265698

[B8] Bednarek A. Cena A. Izak W. Bigos J. Łobocka M. (2022). Functional dissection of P1 bacteriophage holin-like proteins reveals the biological sense of P1 lytic system complexity. Int. J. Mol. Sci. 23:4231. 10.3390/ijms23084231 35457047 PMC9025707

[B9] Bednarek A. Giermasińska-Buczek K. Łobocka M. (2023). Efficient traceless modification of the P1 bacteriophage genome through homologous recombination with enrichment in double recombinants: a new perspective on the functional annotation of uncharacterized phage genes. Front. Microbiol. 14:1135870. 10.3389/fmicb.2023.1135870 37020717 PMC10067587

[B10] Bernard P. (1996). Positive selection of recombinant DNA by CcdB. Biotechniques 21, 320–323. 10.2144/96212pf01 8862819

[B11] Bertani G. (1951). Studies on lysogenesis. I. The mode of phage liberation by lysogenic *Escherichia coli*. J. Bacteriol. 62, 293–300. 10.1128/jb.62.3.293-300.1951 14888646 PMC386127

[B12] Brettin T. Davis J. J. Disz T. Edwards R. A. Gerdes S. Olsen G. J.. (2015). RASTtk: a modular and extensible implementation of the RAST algorithm for building custom annotation pipelines and annotating batches of genomes. Sci. Rep. 5:8365. 10.1038/srep08365 25666585 PMC4322359

[B13] Bulyhina T. V. Zdorovenko E. L. Varbanets L. D. Shashkov A. S. Kadykova A. A. Knirel Y. A.. (2020). Structure of O-polysaccharide and lipid A of *Pantoea agglomerans* 8488. Biomolecules 10:804. 10.3390/biom10050804 32456025 PMC7277085

[B14] Calos M. P. Miller J. H. (1980). Molecular consequences of deletion formation mediated by the transposon Tn*9*. Nature 285, 38–41. 10.1038/285038a0 6246435

[B15] Carobbi A. Di Nepi S. Fridman C. M. Dar Y. Ben-Yaakov R. Barash I.. (2022). An antibacterial T6SS in *Pantoea agglomerans* pv. betae delivers a lysozyme-like effector to antagonize competitors. Environ. Microbiol. 24, 4787–4802. 10.1111/1462-2920.16100 35706135 PMC9796082

[B16] Chandler M. Galas D. J. (1983). Cointegrate formation mediated by Tn9. II. Activity of IS1 is modulated by external DNA sequences. J. Mol. Biol. 170, 61–91. 10.1016/s0022-2836(83)80227-7 6313938

[B17] Chattoraj D. K. Schneider T. D. (1997). Replication control of plasmid P1 and its host chromosome: the common ground. Prog. Nucleic Acid Res. Mol. Biol. 57, 145–186. 10.1016/S0079-6603(08)60280-9 9175433

[B18] Cole S. T. Guest J. R. (1980). Genetic and physical characterization of lambda transducing phages (lambda frdA) containing the fumarate reductase gene of *Escherichia coli* K12. Mol. Gen. Genet. 178, 409–418. 10.1007/BF00270492 6446651

[B19] Coren J. S. Pierce J. C. Sternberg N. (1995). Headful packaging revisited: the packaging of more than one DNA molecule into a bacteriophage P1 head. J. Mol. Biol. 249, 176–184. 10.1006/jmbi.1995.0287 7776370

[B20] Dabrazhynetskaya A. Brendler T. Ji X. Austin S. (2009). Switching protein-DNA recognition specificity by single-amino-acid substitutions in the P1 par family of plasmid partition elements. J. Bacteriol. 191, 1126–1131. 10.1128/JB.01358-08 19028896 PMC2632008

[B21] Dabrazhynetskaya A. Sergueev K. Austin S. (2005). Species and incompatibility determination within the P1par family of plasmid partition elements. J. Bacteriol. 187, 5977–5983. 10.1128/JB.187.17.5977-5983.2005 16109939 PMC1196149

[B22] Davidov E. Kaufmann G. (2008). RloC: a wobble nucleotide-excising and zinc-responsive bacterial tRNase. Mol. Microbiol. 69, 1560–1574. 10.1111/j.1365-2958.2008.06387.x 18681940 PMC2610378

[B23] De Bruijn F. J. Bukhari A. I. (1978). Analysis of transposable elements inserted in the genomes of bacteriophages Mu and P1. Gene 3, 315–331. 10.1016/0378-1119(78)90041-0 365686

[B24] De Coster W. D'Hert S. Schultz D. T. Cruts M. Van Broeckh C. (2018). NanoPack: visualizing and processing long-read sequencing data. Bioinformatics 34, 2666–2669. 10.1093/bioinformatics/bty149 29547981 PMC6061794

[B25] de Lima Procópio R. E. Araújo W. L. Andreote F. D. Azevedo J. L. (2011). Characterization of a small cryptic plasmid from endophytic *Pantoea agglomerans* and its use in the construction of an expression vector. Genet. Mol. Biol. 34, 103–109. 10.1590/S1415-47572010005000096 21637551 PMC3085353

[B26] Delétoile A. Decré D. Courant. S Passet V. Audo J. Grimont P.. (2009). Phylogeny and identification of Pantoea species and typing of *Pantoea agglomerans* strains by multilocus gene sequencing. J. Clin. Microbiol. 47, 300–310. 10.1128/JCM.01916-08 19052179 PMC2643697

[B27] Dubnau D. (1999). DNA uptake in bacteria. Annu. Rev. Microbiol. 53, 217–244. 10.1146/annurev.micro.53.1.217 10547691

[B28] Ershova A. S. Rusinov I. S. Spirin S. A. Karyagina A. S. Alexeevski A. V. (2015). Role of restriction-modification systems in prokaryotic evolution and ecology. Biochemistry 80, 1373–1386. 10.1134/S0006297915100193 26567582

[B29] Evans T. J. Crow M. A. Williamson N. R. Orme W. Thomson N. R. Komitopoulou E.. (2010). Characterization of a broad-host-range flagellum-dependent phage that mediates high-efficiency generalized transduction in, and between, Serratia and Pantoea. Microbiology 156, 240–247. 10.1099/mic.0.032797-0 19778959

[B30] Franklin N. C. (1969). Mutation in galU gene of *E. coli* blocks phage P1 infection. Virology 38, 189–191. 10.1016/0042-6822(69)90144-5 4891220

[B31] Fu J. F. Ying S. W. Liu S. T. (1997). Cloning and characterization of the ori region of pSW1200 of *Erwinia stewartii*: similarity with plasmid P1. Plasmid 38, 141–147. 10.1006/plas.1997.1308 9435016

[B32] Galas D. J. Calos M. P. Miller J. H. (1980). Sequence analysis of Tn*9* insertions in the lacZ gene. J. Mol. Biol. 144, 19–41. 10.1016/0022-2836(80)90213-2 6260963

[B33] Gao L. Altae-Tran H. Böhning F. Makarova K. S. Segel M. Schmid-Burgk J. L.. (2020). Diverse enzymatic activities mediate antiviral immunity in prokaryotes. Science 369, 1077–1084. 10.1126/science.aba0372 32855333 PMC7985843

[B34] Giermasińska K. Łobocka M. (2016). “Interaction of bacteriophage P1 with cells of selected plant pathogens of the genus Erwinia and related genera,” in Selected Issues of Chemistry, Physics and Biology, eds. B. Zdunek and M. Olszówka (Lublin: Tygiel Press), 48–67.

[B35] Gilcrease E. B. Casjens S. R. (2018). The genome sequence of *Escherichia coli* tailed phage D6 and the diversity of Enterobacteriales circular plasmid prophages. Virology 515, 203–214. 10.1016/j.virol.2017.12.019 29304472 PMC5800970

[B36] Goldberg R. B. Bender R. A. Streicher S. L. (1974). Direct selection for P1- sensitive mutants of Enteric Bacteria. J. Bacteriol. 118, 810–814. 10.1128/jb.118.3.810-814.1974 4598005 PMC246826

[B37] Gorb T. E. Kushkina A. I. Ivanitsa T. V. Lysenko T. G. Tovkach F. I. (2011). Structural stability of DNA of the transposon derivatives of pCA25 plasmid. Mikrobiol. Z. 73, 53–57. 21598660

[B38] Grant J. R. Enns E. Marinier E. Mandal A. Herman E. K. Chen C. Y.. (2023). Proksee: in-depth characterization and visualization of bacterial genomes. Nucleic Acids Res. 51, W484–W492. 10.1093/nar/gkad326 37140037 PMC10320063

[B39] Grigoriev P. S. Lobocka M. B. (2001). Determinants of segregational stability of the linear plasmid-prophage N15 of *Escherichia coli*. Mol. Microbiol. 42, 355–368. 10.1046/j.1365-2958.2001.02632.x 11703660

[B40] Haft D. H. DiCuccio M. Badretdin A. Brover V. Chetvernin V. O'Neill K.. (2018). RefSeq: an update on prokaryotic genome annotation and curation. Nucleic Acids Res. 46, D851–D860. 10.1093/nar/gkx1068 29112715 PMC5753331

[B41] Hashimoto M. Ichimura T. Mizoguchi H. Tanaka K. Fujimitsu K. Keyamura K.. (2005). Cell size and nucleoid organization of engineered *Escherichia coli* cells with a reduced genome. Mol. Microbiol. 55, 137–149. 10.1111/j.1365-2958.2004.04386.x 15612923

[B42] Hashimoto M. Satou R. Ozono M. Inagawa H. Soma G. I. (2017). Characterization of the O-antigen polysaccharide derived from *Pantoea agglomerans* IG1 lipopolysaccharide. Carbohydr. Res. 449, 32–36. 10.1016/j.carres.2017.06.017 28686930

[B43] Hazan R. Engelberg-Kulka H. (2004). *Escherichia coli* mazEF-mediated cell death as a defense mechanism that inhibits the spread of phage P1. Mol. Genet. Genom. 272, 227–234. 10.1007/s00438-004-1048-y 15316771

[B44] Huan Y. W. Fa-Arun J. Wang B. (2022). The role of O-antigen in P1 transduction of *Shigella flexneri* and *Escherichia coli* with its alternative S' tail fibre. J. Mol. Biol. 434:167829. 10.1016/j.jmb.2022.167829 36116540

[B45] Huang H. Masters M. (2014). Bacteriophage P1 pac sites inserted into the chromosome greatly increase packaging and transduction of *Escherichia coli* genomic DNA. Virology 468–470, 274–282. 10.1016/j.virol.2014.07.029 25213407

[B46] Humphrey J. Seitz T. Haan T. Ducluzeau A. L. Drown D. M. (2019). Complete genome sequence of *Pantoea agglomerans* TH81, isolated from a permafrost thaw gradient. Microbiol. Resour. Announc. 8, e01486–e01418. 10.1128/MRA.01486-18 30637402 PMC6318373

[B47] Iida S. (1983). On the origin of the chloramphenicol resistance transposon Tn*9*. J. Gen. Microbiol. 129, 1217–1225. 10.1099/00221287-129-4-1217 6310027

[B48] Iida S. Marcoli R. Bickle T. A. (1981). Variant insertion element IS1 generates 8-base pair duplications of the target sequence. Nature 294, 374–376. 10.1038/294374a0 6273737

[B49] Iida S. Streiff M. B. Bickle T. A. Arber W. (1987). Two DNA antirestriction systems of bacteriophage P1, darA, and darB: characterization of darA- phages. Virology 157, 156–166. 10.1016/0042-6822(87)90324-2 3029954

[B50] Il'ina T. S. Nechaeva E. V. Pasynkova L. N. Smirnova N. I. Smirnov G. B. (1980). Tn*9* integration sites and their effect on transposon properties. Genetika 16, 46–54. 6254833

[B51] Johnsrud L. Calos M. P. Miller J. H. (1978). The transposon Tn*9* generates a 9 bp repeated sequence during integration. Cell 15, 1209–1219. 10.1016/0092-8674(78)90047-8 365355

[B52] Jones P. Binns D. Chang H. Y. Fraser M. Li W. McAnulla C.. (2014). InterProScan 5: genome-scale protein function classification. Bioinformatics 30, 1236–1240. 10.1093/bioinformatics/btu031 24451626 PMC3998142

[B53] Kaiser D. Dworkin M. (1975). Gene transfer to myxobacterium by Escherichia coli phage P1. Science 187, 653–654. 10.1126/science.803710 803710

[B54] Karamanos Y. Kol O. Wieruszeski J. M. Strecker G. Fournet B. Zalisz R. (1992). Structure of the O-specific polysaccharide chain of the lipopolysaccharide of *Enterobacter agglomerans*. Carbohydr. Res. 231, 197–204. 10.1016/0008-6215(92)84019-O 1394314

[B55] Kato J. Hashimoto M. (2007). Construction of consecutive deletions of the *Escherichia coli* chromosome. Mol. Syst. Biol. 3:132. 10.1038/msb4100174 17700540 PMC1964801

[B56] Keller C. M. Kendra C. G. Bruna R. E. Craft D. Pontes M. H. (2021). Genetic modification of Sodalis species by DNA transduction. mSphere 6, e01331–e01320. 10.1128/mSphere.01331-20 33597173 PMC8544901

[B57] Kenzaka T. Tani K. Sakotani A. Yamaguchi N. Nasu M. (2007). High-frequency phage-mediated gene transfer among *Escherichia coli* cells, determined at the single-cell level. Appl. Environ. Microbiol. 73, 3291–3299. 10.1128/AEM.02890-06 17384307 PMC1907122

[B58] Kittleson J. T. DeLoache W. Cheng H. Y. Anderson J. C. (2012). Scalable plasmid transfer using engineered P1-based phagemids. ACS Synth. Biol. 1, 583–589. 10.1021/sb300054p 23656280 PMC3804010

[B59] Klaiman D. Steinfels-Kohn E. Krutkina E. Davidov E. Kaufmann G. (2012). The wobble nucleotide-excising anticodon nuclease RloC is governed by the zinc-hook and DNA-dependent ATPase of its Rad50-like region. Nucleic Acids Res. 40, 8568–8578. 10.1093/nar/gks593 22730290 PMC3458546

[B60] Kohchi C. Inagawa H. Nishizawa T. Yamaguchi T. Nagai S. Soma G. (2006). Applications of lipopolysaccharide derived from Pantoea agglomerans (IP-PA1) for health care based on macrophage network theory. J. Biosci. Bioeng. 102, 485–496. 10.1263/jbb.102.485 17270712

[B61] Konddo E. Mitsuhashi S. (1964). Drug resistance of enteric bacteria. IV. Active transducing bacteriophage P1 CM produced by the combination of R factor with bacteriophage P1. J. Bacteriol. 88, 1266–1276. 10.1128/jb.88.5.1266-1276.1964 14234780 PMC277403

[B62] Lam C. N. Mehta-Kolte M. G. Martins-Sorenson N. Eckert B. Lin P. H. Chu K.. (2021). A tail fiber engineering platform for improved bacterial transduction-based diagnostic reagents. ACS Synth. Biol. 10, 1292–1299. 10.1021/acssynbio.1c00036 33983709

[B63] Lee S. I. Tran T. D. Huynh S. Parker C. T. Hnasko R. McGarvey J. A. (2021). Complete genome sequence of *Pantoea agglomerans* ASB05 using illumina and PacBio sequencing. Microbiol. Resour. Announc. 10:e0050121. 10.1128/MRA.00501-21 34323608 PMC8320467

[B64] Lennox E. S. (1955). Transduction of linked genetic characters of the host by bacteriophage P1. Virology 1, 190–206. 10.1016/0042-6822(55)90016-7 13267987

[B65] Lerouge I. Vanderleyden J. (2002). O-antigen structural variation: mechanisms and possible roles in animal/plant-microbe interactions. FEMS Microbiol. Rev. 26, 17–47. 10.1111/j.1574-6976.2002.tb00597.x 12007641

[B66] Lindow S. E. Desurmont C. Elkins R. McGourty G. Clark E. Brandl M. T. (1998). Occurrence of indole-3-acetic acid-producing bacteria on pear trees and their association with fruit russet. Phytopathology 88, 1149–1157. 10.1094/PHYTO.1998.88.11.1149 18944847

[B67] Liu J. Chen C. Y. Shiomi D. Niki H. Margolin W. (2011). Visualization of bacteriophage P1 infection by cryo-electron tomography of tiny *Escherichia coli*. Virology 417, 304–311. 10.1016/j.virol.2011.06.005 21745674 PMC3163801

[B68] Łobocka M. Gągała U. (2021). “Prophage P1: an example of a prophage that is maintained as a plasmid,” in Bacteriophages, eds D. R. Harper, S. T. Abedon, B. H. Burrowes, and M. L. McConville (Cham: Springer). 10.1007/978-3-319-40598-8_54-1

[B69] Łobocka M. Yarmolinsky M. (1996). P1 plasmid partition: a mutational analysis of ParB. J. Mol. Biol. 259, 366–382. 10.1006/jmbi.1996.0326 8676375

[B70] Łobocka M. B. Rose D. J. Plunkett G. Rusin M. Samojedny A. Lehnherr H.. (2004). Genome of bacteriophage P1. J. Bacteriol. 186, 7032–7068. 10.1128/JB.186.21.7032-7068.2004 15489417 PMC523184

[B71] Lorenzi A. S. Bonatelli M. L. Chia M. A. Peressim L. Quecine M. C. (2022). Opposite sides of *Pantoea agglomerans* and its associated commercial outlook. Microorganisms 10:2072. 10.3390/microorganisms10102072 36296348 PMC9610544

[B72] Machida C. Machida Y. Wang H. C. Ishizaki K. Ohtsubo E. (1983). Repression of cointegration ability of insertion element IS1 by transcriptional readthrough from flanking regions. Cell 34, 135–142. 10.1016/0092-8674(83)90143-5 6309405

[B73] Matilla M. A. Evans T. J. Martín J. Udaondo Z. Lomas-Martínez C. Rico-Jiménez M.. (2023). Herbicolin A production and its modulation by quorum sensing in a *Pantoea agglomerans* rhizobacterium bioactive against a broad spectrum of plant-pathogenic fungi. Microb. Biotechnol. 16, 1690–1700. 10.1111/1751-7915.14193 36528875 PMC10364316

[B74] Meyer J. Iida S. Arber W. (1980). Does the insertion element IS1 transpose preferentially into A+T-rich DNA segments? Mol. Gen. Genet. 178, 471–473. 10.1007/BF00270502 6248730

[B75] Miller J. H. (1972). Experiments in Molecular Genetics. Cold Spring Harbor, NY: Cold Spring Harbor Lab Press.

[B76] Miller J. H. Calos M. P. Galas D. Hofer M. Büchel D. E. Müller-Hill B. (1980). Genetic analysis of transpositions in the lac region of *Escherichia coli*. J. Mol. Biol. 144, 1–18. 10.1016/0022-2836(80)90212-0 6260962

[B77] Millman A. Melamed S. Leavitt A. Doron S. Bernheim A. Hör J.. (2022). An expanded arsenal of immune systems that protect bacteria from phages. Cell Host Microbe. 30, 1556–1569.e5. 10.1016/j.chom.2022.09.017 36302390

[B78] Murooka Y. Harada T. (1979). Expansion of the host range of coliphage P1 and gene transfer from enteric bacteria to other gram-negative bacteria. Appl. Environ. Microbiol. 38, 754–757. 10.1128/aem.38.4.754-757.1979 395900 PMC243574

[B79] Novick R. P. (1987). Plasmid incompatibility. Microbiol. Rev. 51, 381–395. 10.1128/mr.51.4.381-395.1987. 3325793 PMC373122

[B80] Ohtsubo H. Ohtsubo E. (1978). Nucleotide sequence of an insertion element, IS1. Proc. Natl. Acad. Sci. U. S. A. 75, 615–619. 10.1073/pnas.75.2.615 273224 PMC411306

[B81] Ornellas E. P. Stocker B. A. (1974). Relation of lipopolysaccharide character to P1 sensitivity in *Salmonella typhimurium*. Virology 60, 491–502. 10.1016/0042-6822(74)90343-2 4602344

[B82] Overbeek R. Olson R. Pusch G. D. Olsen G. J. Davis J. J. Disz T.. (2014). The SEED and the Rapid Annotation of microbial genomes using Subsystems Technology (RAST). Nucleic Acids Res. 42, D206–D214. 10.1093/nar/gkt1226 24293654 PMC3965101

[B83] Radnedge L. Youngren B. Davis M. Austin S. (1998). Probing the structure of complex macromolecular interactions by homolog specificity scanning: the P1 and P7 plasmid partition systems. EMBO J. 17, 6076–6085. 10.1093/emboj/17.20.6076 9774351 PMC1170934

[B84] Rekosz-Burlaga H. Borys M. Goryluk-Salmonowicz A. (2014). Cultivable microorganisms inhabiting the aerial parts of *Hypericum perforatum*. Acta Scientiar. Polonor. Hortor. Cult. 13, 117–129.

[B85] Ren J. Karna S. Lee H. M. Yoo S. M. Na D. (2019). Artificial transformation methodologies for improving the efficiency of plasmid DNA transformation and simplifying its use. Appl. Microbiol. Biotechnol. 103, 9205–9215. 10.1007/s00253-019-10173-x 31650193

[B86] Résibois A. Toussaint A. van Gijsegem F. Faelen M. (1981). Physical characterization of mini-Mu and mini-D108. Gene 14, 103–113. 10.1016/0378-1119(81)90152-9 6266926

[B87] Rosner J. L. (1972). Formation, induction, and curing of bacteriophage P1 lysogens. Virology 48, 679–689. 10.1016/0042-6822(72)90152-3 4555608

[B88] Sambrook J. Fritsch E. F. Maniatis T. (1989). Molecular Cloning: A Laboratory Manual. Cold Spring Harbor, NY: Cold Spring Harbor Laboratory.

[B89] Sergueev K. Dabrazhynetskaya A. Austin S. (2005). Plasmid partition system of the P1par family from the pWR100 virulence plasmid of *Shigella flexneri*. J. Bacteriol. 187, 3369–3373. 10.1128/JB.187.10.3369-3373.2005 15866921 PMC1112009

[B90] Shetty S. Kamble A. Singh H. (2023). Insights into the potential role of plasmids in the versatility of the genus Pantoea. Mol. Biotechnol. 2023:3. 10.1007/s12033-023-00960-3 38007817

[B91] Smits T. H. Rezzonico F. Kamber T. Goesmann A. Ishimaru C. A. Stockwell V. O.. (2010). Genome sequence of the biocontrol agent *Pantoea vagans* strain C9-1. J. Bacteriol. 192, 6486–6487. 10.1128/JB.01122-10 20952567 PMC3008540

[B92] Sternberg N. L. Maurer R. (1991). Bacteriophage-mediated generalized transduction in *Escherichia coli* and *Salmonella typhimurium*. Methods Enzymol. 204, 18–43. 10.1016/0076-6879(91)04004-8 1943777

[B93] Streiff M. B. Iida S. Bickle T. A. (1987). Expression and proteolytic processing of the darA antirestriction gene product of bacteriophage P1. Virology 157, 167–171. 10.1016/0042-6822(87)90325-4 3029955

[B94] Surtees J. A. Funnell B. E. (2003). Plasmid and chromosome traffic control: how ParA and ParB drive partition. Curr. Top. Dev. Biol. 56, 145–180. 10.1016/S0070-2153(03)01010-X 14584729

[B95] Tatusova T. DiCuccio M. Badretdin A. Chetvernin V. Nawrocki E. P. Zaslavsky L.. (2016). NCBI prokaryotic genome annotation pipeline. Nucleic Acids Res. 44, 6614–6624. 10.1093/nar/gkw569 27342282 PMC5001611

[B96] Tesson F. Hervé A. Mordret E. Touchon M. d'Humières C. Cury J.. (2022). Systematic and quantitative view of the antiviral arsenal of prokaryotes. Nat. Commun. 13:2561. 10.1038/s41467-022-30269-9 35538097 PMC9090908

[B97] Thissera B. Alhadrami H. A. Hassan M. H. A. Hassan H. M. Bawazeer M. Yaseen M.. (2020). Induction of cryptic antifungal pulicatin derivatives from *Pantoea agglomerans* by microbial co-culture. Biomolecules 10:268. 10.3390/biom10020268 32050703 PMC7072716

[B98] Thomason L. C. Costantino N. Court D. L. (2007). *E. coli* genome manipulation by P1 transduction. Curr. Protoc. Mol. Biol. 79, 1.17.1–1.17.8. 10.1002/0471142727.mb0117s79 18265391

[B99] Tominaga A. Enomoto M. (1986). Magnesium-dependent plaque formation by bacteriophage P1cinC(-) on *Escherichia coli* C and *Shigella sonnei*. Virology 155, 284–288. 10.1016/0042-6822(86)90190-X 3535235

[B100] Varbanets L. D. Bulyhina T. V. Pasichnyk L. A. Zhytkevich N. V. (2019). *Pantoea agglomerans* lipopolysaccharides: structure, functional and biological activity. Ukr. Biochem. J. 91, 5–20. 10.15407/ubj91.01.00530141844

[B101] Walker J. T. Walker D. H. Jr. (1981). Structural proteins of coliphage P1. Prog. Clin. Biol. Res. 64, 69–77. 7330064

[B102] Walterson A. M. Stavrinides J. (2015). Pantoea: insights into a highly versatile and diverse genus within the Enterobacteriaceae. FEMS Microbiol. Rev. 39, 968–984. 10.1093/femsre/fuv027 26109597

[B103] Westwater C. Schofield D. A. Schmidt M. G. Norris J. S. Dolan J. W. (2002). Development of a P1 phagemid system for the delivery of DNA into Gram-negative bacteria. Microbiology 148, 943–950. 10.1099/00221287-148-4-943 11932441

[B104] Williams A. N. Sorout N. Cameron A. J. Stavrinides J. (2020). The integration of genome mining, comparative genomics, and functional genetics for biosynthetic gene cluster identification. Front. Genet. 11:600116. 10.3389/fgene.2020.600116 33343637 PMC7744662

[B105] Wilson K. (2001). Preparation of genomic DNA from bacteria. Curr. Protoc. Mol. Biol. 2:2.4. 10.1002/0471142727.mb0204s56 18265184

[B106] Wilson M. Lindow S. E. (1994). Coexistence among epiphytic bacterial populations mediated through nutritional resource partitioning. Appl. Environ. Microbiol. 60, 4468–4477. 10.1128/aem.60.12.4468-4477.1994 16349462 PMC202007

[B107] Wright S. A. Zumoff C. H. Schneider L. Beer S. V. (2001). *Pantoea agglomerans* strain EH318 produces two antibiotics that inhibit *Erwinia amylovora in vitro*. Appl. Environ. Microbiol. 67, 284–292. 10.1128/AEM.67.1.284-292.2001 11133457 PMC92566

[B108] Xu S. Liu Y. X. Cernava T. Wang H. Zhou Y. Xia T.. (2022). Fusarium fruiting body microbiome member *Pantoea agglomerans* inhibits fungal pathogenesis by targeting lipid rafts. Nat. Microbiol. 7, 831–843. 10.1038/s41564-022-01131-x 35618775

[B109] Yamazaki Y. Niki H. Kato J. (2008). Profiling of *Escherichia coli* chromosome database. Methods Mol. Biol. 416, 385–389. 10.1007/978-1-59745-321-9_26 18392982

[B110] Yarmolinsky M. Sternberg N. (1988). “Bacteriophage Pl,” in The Bacteriophages, vol. 1, ed. R. Calendar (New York, NY: Plenum Press), 291–438.

[B111] Yarmolinsky M. B. (2004). Bacteriophage P1 in retrospect and prospect. J. Bacteriol. 186, 7025–7028. 10.1128/JB.186.21.7025-7028.2004 15489415 PMC523185

[B112] Yarmolinsky M. B. Hoess R. (2015). The legacy of Nat Sternberg: the genesis of Cre-lox technology. Annu. Rev. Virol. 2, 25–40. 10.1146/annurev-virology-100114-054930 26958905

