# Supplementary material for: Interaction of bacteriophage P1 with an epiphytic Pantoea agglomerans strain—the role of the interplay between various mobilome elements
Source: Front Microbiol. 2024 Mar 25;15:1356206. doi: 10.3389/fmicb.2024.1356206 (PMC10999674; doi:10.3389/fmicb.2024.1356206)
Supplement: Supplementary file 1 [file Data_Sheet_1.pdf]

frontiers

Supplementary Material

Interaction of bacteriophage P1 with an epiphytic *Pantoea agglomerans* strain - the role of the interplay between various mobilome elements

Katarzyna Giermasińska-Buczek[1,2,], Jan Gawor[2], Emil Stefańczyk[2], Urszula Gągała[1], Karolina Żuchniewicz[2], Hanna Rekosz-Burlaga[1], Robert Gromadka[2] and Małgorzata Łobocka[2]

[1]Department of Biochemistry and Microbiology, Institute of Biology, Warsaw University of Life Sciences (SGGW-WULS), Warsaw, Poland

[2] Institute of Biochemistry and Biophysics of the Polish Academy of Sciences, Warsaw, Poland

* **Correspondence: Małgorzata Łobocka:** lobocka@ibb.waw.pl

1 Supplementary Experimental Procedures

1.1. Phage adsorption

Phage adsorption experiments were carried out as described previously (Głowacka-Rutkowska et al., 2019) with some modifications. Overnight cultures of *E. coli* N99 and *P. agglomerans* L15 cells grown in LB liquid medium, at 30 °C were refreshed in similar medium and grown with shaking to the optical density (OD_{600}) of about 0.4. Aliquots of each culture (500 µl) were supplemented with 250 µl of 20 mM CaCl2, 250 µl 20 mM MgSO, and 500 µl of lysate containing phages to obtain the multiplicity of infection (M.O.I.) £ 1, and incubated at room temperate for various 5, 10, and 15 min. without shaking. At the end of a given incubation time samples were immediately filtered through 0.20 µm syringe filters (Filtropur S, cat. no. 83.1825.001; Sarstedt, Nümbrecht, Germany) and used to determine the titer of unadsorbed phages by the double layer agar method (Adams, 1959). The fraction of adsorbed phages (in %) was calculated by the subtraction of the titer of unadsorbed phages from the initial titer of infecting phages, relative to the phage initial titer.

1.2. Optimization of P1-mediated plasmid transduction from *E. coli* to *P. agglomerans* L15

The region of P1 *c1-100* Tn*9* genome permissible for the insertion of about 12 kb plasmid without compromisig phage functionality was selected based on the ability of P1 *c1-100* Tn*9* obtained by the induction of lysogens of *E. coli* containing derivatives of a wide-host range pRK2

plasmid with cloned fragments of various P1 genome regions, to transduce these plasmids (see Table S1 and S3). In the first step, the *E. coli* C600 cells with any of these plasmids were lysogenized with P1 *c1-100* Tn*9.* Lysogens obtained as able to grow on solid medium with chloramphenicol (selective for P1 *c1-100* Tn*9)*, and tetracycline (selective for the pRK2-derived plasmids), were used to inoculate LB medium with chloramphenicol and tetracycline to induce the lytic development of prophage P1 *c1-100* Tn*9.* The phages (10^8 and 10^6 pfu/mL) obtained through the thermal induction of lysogens served to infect cells of the *E. coli* C600 strain. Infected cells that could form colonies on LA medium with tetracycline, but without chloramphenicol at 30 °C, served to isolate plasmid DNA and verify, by restriction digestion, the identity of obtained plasmid with the plasmid present in the donor cells (Table S3, Figure S2). Only in the case of phage that was propagated in cells with the pKGI5 (*phddoc*$^+$) plasmid single Tc^RCm^S colonies were obtained and they carried the pKGI5 plasmid. Thus, a cell infected with this phage and likely to contain a hybrid of P1 *c1-100* Tn*9* prophage and pKGI5 plasmid, as verified by its ability to grow on medium with chloramphenicol and tetracycline, and by restriction digestion of its plasmid DNA (Figure S2) was used to induce the lytic development of hybrid P1. Phages of the obtained lysate were used for transduction of the *E. coli* C600 cells from cultures of different optical densities (OD_{600} 1.4 and 0.3) and with the use of different MOIs. Conditions optimal for the transduction of pKGI5 plasmid to *E. coli* cells (OD600 of recipient cell culture of about 0.3, MOI of about 10^5) were used for transduction of *P. agglomerans* L15 (Table S4). The presence of pKGI5 plasmid in L15 transformants grown on LA medium with tetracycline, but sensitive to chloramphenicol was verified based on the restriction digestion pattern of plasmid DNA isolated from them and by testing whether plasmid DNA isolated from them can serve as a template for PCR amplification with primers specific for various regions of pKGI5 (Figure S3).

1.3. Shotgun diagnostic WGS sequencing

Diagnostic long-read sequencing of selected isolates of *P. agglomerans* L15 or its derivatives cured from plasmids or lysogenized with P1 *c1-100* Tn*9* IS*1*::km^R was performed using MinION instrument (Oxford Nanopore Technologies, Oxford, UK). Nanopore libraries were constructed using Rapid Barcoding SQK-RBK004 kit and sequenced on MinION R9.4.1 flowcell. Raw nanopore reads were basecalled using guppy v.6.5.7 (Oxford Nanopore Technologies, Oxford, UK). Sequencing adapter removal was performed using Porechop (https://github.com/rrwick/Porechop) and quality filtering was done (mean read quality of 10 and read length of 1kb) using filtlong (https://github.com/rrwick/Filtlong). Cleaned nanopore reads were mapped to reference sequences set consisting of L15 strain plasmids sequences and P1 bacteriophage genome using minimap2 (Li, 2018). Alignment files were converted to sorted and indexed bam alignment files using samtools v1.6 (Danecek et al., 2021). Coverage statistics were calculated using Qualimap bamqc tool (http://qualimap.conesalab.org/).

1.4. Assembly of bacteriophage P1 genome from long nanopore reads.

Nanopore reads from L15 strains containing bacteriophage P1 replicon were merged into one fastq file and mapped to P1 reference sequence using minimap2. Sequence coverage statistics were

calculated using Qualimap bamqc tool. All reads that uniquely mapped to reference sequence were extracted from bam alignment file using samtools v1.6 (Danecek et al., 2021). Obtained long reads were then used for *de novo* assembly of P1 *c1-100* Tn*9* IS*1*::km^R genome using flye assembler (Kolomogorov, 2019).

2 Supplementary Figures and Tables

2.1. Tables

Supplementary Table 1. Plasmids used in this study

Plasmid[a]	Cloned genes	Description (comments)	Replication origin /Selective marker	Source or reference
pUC4K	km^R	A multicopy *E. coli* vector containing the kanamycin resistance cassette of Tn*903*	pMB1/ap^R, km^R	Taylor and Rose,1988
pUC18	-	Standard *E. coli* vector with a multiple cloning site (MCS) for DNA cloning	pMB1/ap^R	Vieira and Messing, 1982
pUCP1/270	IS*1*	A derivative of pUC18 plasmid vector with a cloned fragment of P1 genomic library containing the 3' fragment *isaA* gene, and the 5' fragment of IS*1* sequence (pos. 22011+/-10- do 23306+/-10 of the P1 genome)	pMB1/ap^R	Plasmid collection of IBB PAS; Łobocka et al., 2004
pRK415	tc^R	A mini-RK2 derived low copy number vector containing the tcracycline resistance cassette	RK2/tc^R	(Keen et al., 1988)
pKGI2	*pdcB* ::km^R	A derivative of pUC18 plasmid with cloned *pdcB* gene of bacteriophage P1 inactivated by the insertion of kanamycin resistance cassette	pMB1/ap^R	Bednarek et al., 2023
pPagL15_1	-	Natural plasmid of *P. agglomerans* L15		This study
pPagL15_2	-	Natural plasmid of *P. agglomerans* L15		This study
pPagL15_3	-	Natural plasmid of *P. agglomerans* L15		This study
pKGI5	*phd doc*	A derivartive of pRK415 in which the PstI-PstI fragment (pos. 893-1759) was replaced with a 512-bp fragment containing the *phd* and *doc* genes of bacteriophage P1. The P1 DNA fragment containing the *phd* and *doc* genes was amplified with the use of OMLO755 and OMLO756 primers and bacteriophage P1 DNA as a template.	RK2/tc^R	This study
pKGI6	*mod*	A derivartive of pRK415 in which the HindIII-HindIII fragment (pos. 901-1743) was replaced with a 649-bp fragment containing the *mod* gene of bacteriophage P1. The P1 DNA fragment containing the *mod* gene was amplified with the use of OMLO757 and OMLO758 primers and bacteriophage P1 DNA as a template.	RK2/tc^R	This study

pKGI7	IS*1*	A derivartive of pRK415 in which the HindIII-HindIII fragment (pos. 901-1743) was replaced with a 570-bp fragment containing the IS*1* sequence of bacteriophage P1. The P1 DNA fragment containing the IS*1* sequence was amplified with the use of OMLO759 and OMLO760 primers and bacteriophage P1 DNA as a template.	RK2/tc^R	This study
pKGI10	km^R, IS*1*	A derivartive of pUCP1/270 containing the km^R cassette in the IS*1* sequence of bacteriophage P1 (at pos. 322 of IS*1*). obtained by the insertion of the km^R cassette of pUC4K plasmid amplified with OMLO623 and OMLO797 primers and digested with MluII into the MluI site of pUCP1/270 IS*1* sequence.	pMB1/ap^R, km^R	This study
pKGI12	*ccdA*	A derivartive of pUCP1/270 containing the *ccdA* gene of the *P. agglomerans* L15 pPagL15_3 plasmid inserted in the IS*1* sequence of bacteriophage P1 (pos. 322), and obtained by the insertion of *ccdA* gene amplified with OMLO806 and OMLO807 primers and digested with MluII in the MluI site of the pUCP1/270 IS*1* sequence.	pMB1/ap^R	This study

Supplementary Table 2. Oligonucleotides used in this work

Name	Sequence 5'-3'	Types of primers	Complementary region	Aim of use	Product length (bp)	Tm [°C]
OMLO 531	GCGTAAGCGCCCGGGTATGT	F	*P. agglomerans* L15 genome, pos. 4334-4354	Amplification of *gyrB* gene of *P. agglomerans* (species identification)	417	60ºC
OMLO 532	CCGTCGACGTCCGCATCGGTCAT	R	*P. agglomerans* L15 genome, pos. 5819-5841			
OMLO 533	GATGTGGCGTGTTACGGTGAA	F	Transposon Tn*9*, pos.1298-1318	Amplification of *cat* gene of Tn*9* (verification of the transposon Tn*9* presence in the genome of P1 and *P. agglomerans*)	282	55ºC
OMLO 534	CTGCCACTCATCGCAGTACTGTT	R	Transposon Tn*9*, pos. 1557-1579			
OMLO 738	ATCGGCACGTAAGAGGTTCCAACTTTC	F	Transposon Tn*9*, pos. 831-857	Confirmation of the transposon Tn*9* presence in a phage P1 and *P. agglomerans* L15 genome (after infection with phage P1) by sequencing	1000	55°C
OMLO 739	CGGTATCAACAGGGACACCAGGATTTA	R	Transposon Tn*9*, pos. 765-791			
OMLO 755	ATTCTGCAGGCAATCCATTAACTTCCGTA	F	Phage P1 *c1-100 mod749*::IS5 IS*1*::Tn*9* genome, pos. 88048-88067	Amplification of *phd* and *doc* genes of phage P1 and addition of PstI recognition sites at its flanks	512	55°C
OMLO 756	ATTCTGCAGGCCAGTTCAGGTGAATCAA	R	Phage P1 *c1-100 mod749*::IS5 IS*1*::Tn*9* genome,			

			pos. 88541-88559			
OMLO 757	ATTAAGCTTCTCGGCTG ACTCAGTCATTTCA	F	Phage P1 *c1-100 mod749*::IS*5* IS*1*::Tn*9* genome, pos. 7681-7702	Amplification of *mod* gene of phage P1 and addition of HindIII recognition sites at its flanks	649	57,5°C
OMLO 758	ATTAAGCTTCAGCTC-GACCAAGAACAAAGAG	R	Phage P1 *c1-100 mod749*::IS*5* IS*1*::Tn*9* genome, pos. 8308-8329			
OMLO 759	ATTAAGCTTTGCCAGGT GGTGCCTCAGATTC	F	Phage P1 *c1-100 mod749*::IS*5* IS*1*::Tn*9* genome, pos. 22734-22755	Amplification of IS*1* sequence of phage P1 and addition of HindIII recognition sites at its flanks	570	60°C
OMLO 760	ATTAAGCTTAGCTACTG ACGGGGTGGTGCGTA	R	Phage P1 *c1-100 mod749*::IS*5* IS*1*::Tn*9* genome, pos. 23281-23303			
OMLO 763	AACTTTTGGCGAAAATG AGACGTTGATCGG	F	Transposon Tn*9*, pos. 806-835	The region preceding the *cat* gene of the transposon Tn*9*. Identification of the location of the transposon Tn*9* in the genome of phage P1 by sequencing	-	65°C
trfAF	GTGAAGATCACCTACAC CGGC	F	pRK415 plasmid, pos. 3500-3520	Amplification of *trfA* and *trfB* genes of pRK415 plasmid	130	55°C
trfAR	TGGCAAAGCTCGTAGA ACGTG	R	pRK415 plasmid, pos. 3390-3410			
OMLO 623	ATTACGCGTGCGCTGAG GTCTGCCTCGTGAAGA	F	pUC4K plasmid, pos. 1-24	Amplification of Kan^R cassette of pUC4K plasmid and addition of MluI recognition sites at its flanks	1213	54°C
OMLO 797	ATTACGCGTAAAGCCAC GTTGTGTCTCAAAATC	R	pUC4K plasmid, pos. 1190-1213			
OMLO 806	ATTACGCGTTCCGTGTG ATTGGCAGTGTTAGT	F	pPagL15_3 plasmid of *P. agglomerans* L15, pos. 22085-22107	Amplification of *ccdA* gene of pPagL15_3 plasmid and addition of MluI recognition sites at its flanks	353	56,5°C
OMLO 807	ATTACGCGTGTAACATC GAGCAGGGGATA	R	pPagL15_3 plasmid of *P. agglomerans* L15, pos. 21755-21777			
OMLO 919	AATAGCTAGCGTGCTTA AACAGAGAATGCC	F	Phage P1 *c1-100 mod749*::IS*5* IS*1*::Tn*9* genome, pos. 88799-88818	Amplification of *pdcB* gene of phage P1 and addition of NheI and XhoI recognition sites at its flanks	1109	54,6 °C
OMLO 920	ATACTCGAGCAATTTTAT CTAACACTCGACG	R	Phage P1 *c1-100 mod749*::IS*5* IS*1*::Tn*9* genome, pos. 89886-89907			
OMLO 921	GAAAATGATGAGTGAG TCTGAC	F	pPagL15_3 plasmid of *P. agglomerans* L15, pos. 2271-	Amplification of *parB* gene of pPagL15_3 plasmid	928	54,4°C

			2292			
OMLO 922	GTCTTTTTCATTCACAA CCA	R	pPagL15_3 plasmid of *P. agglomerans* L15, pos. 1365-1384			
OMLO 952	ATTAAGCTTACTGCATT CTTAAAATGTCC	F	pPagL15_3 plasmid of *P. agglomerans* L15, pos. 21813-21832	Amplification of *ccdA* gene of pPagL15_3 plasmid and addition of HindIII and SalI recognition site at its flanks	407	56°C
OMLO 934	ATTGTCGACGTAGTTCC TGTATTTCCATCA	R	pPagL15_3 plasmid of *P. agglomerans* L15, pos. 22199-22219			

[a]Positions in the genomes of plasmid pUC4K, pPagL15_3 and phage P1 *c1-100 mod749*::IS*5* IS*1*::Tn*9* refer to the sequences deposited in GenBank under the accession numbers X06404.1, CP034151, AF234172.1, respectively.

Supplementary Table 3. The number of *E. coli* C600 colonies grown on media with various antibiotics after infection with phages obtained by induction of P1 *c1-100* Tn*9* lysogens of *E. coli* N99 cells carrying plasmids with various fragments of the P1 genome

**Plasmid present in a phage propagation host (P1 genome region cloned)**	**Phage titer in lysate**	**Number of colonies* grown in LA medium with:**		
		**Cm**	**Tc**	**Cm Tc**
**pKGI5 (*phddoc*)**	10^8	uncountable	23	6
	10^6	uncountable	4	0
**pKGI6 (*mod*)**	10^8	uncountable	0	0
	10^6	uncountable	0	0
**pKGI7 (IS*1*)**	10^8	uncountable	0	0
	10^6	uncountable	0	0

* The numbers shown represent the average number of colonies obtained in four independent biological repetitions of each experiment.

Supplementary Table 4. The number of *E. coli* C600 or *P. agglomerans* L15 colonies grown on media with various antibiotics after infection with phages obtained by the induction of *E. coli* C600 lysogen carrying the P1 *c1-100* Tn*9*-pKGI5 hybrid prophage

Target cells	Culture density of infected cells	Phage titer in lysate	Number of colonies grown on LA medium with:		
			**Cm**	**Tc**	**Cm Tc**
E. coli C600	1.4	10^7	uncountable	40	0
		10^5	uncountable	134	5
	0.3	10^7	uncountable	156	2
		10^5	uncountable	432	5
P. agglomerans L15*		10^7	uncountable	0	0
		10^5	492	2	0

*Phage infection of *P. agglomerans* L15 was performed with the use of 10 times increased volumes of the culture and the lysate as compared to those used for the infection of *E. coli* C600.

Supplementary Table 5. *P. agglomerans* L15 genes encoding proteins significantly similar to the proteins of known bacterial anti-phage defense systems

L15 genome part (GenBank acc . no)	System ID[1]	Type/ subtype	Start coord.	End coord.	Num-ber of genes	Protein coding gene starts coord. in the system	N-terminal protein sequences	Profile name in the system
chromosome(CP034148)	Mokosh_TypeII_1	Mokosh/ Mokosh_TypeII	541254	541254	1	541254	MDSNTQGFTS	Mokosh_TypeII_MkoC
	Shango_2	Shango/ Shango	3084537	3088052I	3	3084537 3086732, 3088052	MSSAYDSLDP MSLTRIRVKE MELWLFLAAI	Shango_SngC, Shango_SngB, Shango__SngA
	RloC_1	RloC/RloC	2830219	2830219	1	2830219	MSQLEIRGVR	RloC__RloC
	RM_Type_I_1	RM/ RM_Type_I	530103	537360	4	530103, 531608, 534220, 537360	MTLINLKDLE MSNKKLEEIL MFNEQTVTEN MNTSLFEDLL	RM_Type_I_Mtases, RM_Type_I_Mtases, RM_Type_I_Reases, RM_Type_I_MTases
	MazEF	TA	1722182	1722766	2	1722182 1722518	MVSRFVPDAG MIHGNVKRWG	MazE antitoxin MazF toxin
pPagL15_1 (CP034149)	RM_Type_II_1	RM/ RM_Type_II	481968	483943	2	481968, 483943	MVNKLYLNFH MKAIDLFCGA	RM_Type_II_Type_II_Reases, RM_Type_II_Type_II_MTases
pPagL15_3 (CP034151)	Gao_Ppl_1	Gao_Ppl/ Gao_Ppl	11414	11414	1	11414	MSVGSRWYKF	Gao_Ppl__PplA

Supplementary Table 6. Predicted proteins of *P. agglomerans* L15 significantly similar to bacteriophage P1 proteins

P1 protein	Number of amino acid residues	Predicted product of L15 chromosome (number of amino acid residues), protein ID	% coverage/ % identity	Predicted product of pPagL15_1 plasmid (number of amino acid residues), protein ID	% coverag e/% identity	Predicted product of pPagL15_2 plasmid (number of amino acid residues), protein ID	% coverage/ % identity	Predicted product of pPagL15_3 plasmid (number of amino acid residues) , protein ID	% coverag e/% identity
Ssb	162	549.44.peg.3044 (187) AZI52200.1	100/60						
HrdC	301	549.44.peg.2480 (303) AZI51656.1	98/59						
Ban	454	549.44.peg.3052 (468) AZI52206.1	97/78						
Ppp	230	549.44.peg.1190 (215) AZI50471.1	93/48						
Hot	87	549.44.peg.1161 (95) AZI50444.1	80/60						
HumD	129	549.44.peg.1603 (139) AZI50853.1	89/46						
ParB	333			549.45.peg.568 (323) WP_124890719.1	98/53	549.46.peg.69 (323) WP_124890785.1	94/42	549.47.peg.4 (324) WP_124890853.1	95/43
ParA	398			549.45.peg.569 (399) WP_010246822.1	99/78	549.46.peg.70 (400) WP_124890787.1	97/58	549.47.peg.5 (402) WP_069026998.1	97/54
RepA	286			549.45.peg.571 (309) WP_010246820.1	91/49	549.46.peg.71 (316) WP_010256943.1	88/47	549.47.peg.9 WP_124890855.1	98/73

*Only proteins with over 40% identity to P1 proteins over at least 80% of their length were considered as significantly similar.

Supplementary Table 7. Detection of plasmid or prophage DNA by diagnosting long-read WGS sequencing in cells of *P. agglomerans* L15 or its derivatives cured of particular plasmids or lysogenized with bacteriophage P1 *c1-100* Tn*9* IS*1*::km^{R}

Strain	Reference sequence	Length (bp)	Mapped bases	Mean coverage	Standard deviation	Match to reference
***P. agglomerans* L15**	PpagL15_1	583571	4515151	7.7371	3.5398	YES
	PpagL15_2	179590	1076018	5.9915	2.6126	YES
	PpagL15_3	66484	275114	4.138	1.2644	YES
	Bacteriophage P1	94800	0	0	0	NO
***P. agglomerans* IPAG312**	PpagL15_1	583571	31188	0.0534	0.7287	NO*
	PpagL15_2	179590	516105	2.8738	1.476	YES
	PpagL15_3	66484	145246	2.1847	1.3995	YES
	Bacteriophage P1	94800	0	0	0	NO
***P. agglomerans* IPAG312 cured of pPagL15_3 using P1 *c1-100* Tn*9* IS*1*::km^{R} -mediated replication incompatibility**	PpagL15_1	583571	35863	0.0615	0.7753	NO*
	PpagL15_2	179590	796824	4.4369	1.8461	YES
	PpagL15_3	66484	0	0	0	NO
	Bacteriophage P1	94800	265315	2.7987	1.5473	YES
***P. agglomerans* L15 cured of pPagL15_1 and pPagL15_3 using P1 *c1-100* Tn*9* IS*1*::km^{R} -mediated partition and replication incompatibility, respectively**	PpagL15_1	583571	68320	0.1171	1.2518	NO*
	PpagL15_2	179590	705034	3.9258	2.2059	YES
	PpagL15_3	66484	0	0	0	NO
	Bacteriophage P1	94800	499866	5.1	1.7937	YES
**Bacteriophage P1 *c1-100* Tn*9* IS*1*::km^{R} de novo assembly from the L15 derivatives cured of pPagL15_1 and pPagL15_3**	NA	97417	876048	8.9928	2.6203	NA

*Matches to the reference pPagL15_1 sequence are slightly above 0 due to the presence of homologs of certain chromosomal genes in the sequence of pPagL15_1;
NA - not applicable

Supplementary Table 8. *Pantoea* sp. plasmids partitionally incompatible with P1, by prediction, based on the similarity of their ParB proteins to ParB of pPagL15_1 and P1, and on the conservation of ParB amino acid residues essential for specific interaction with P1 ParB.

Description	Scientific name	Max score	Total score	Query cove-rage	E -value	Identity (%)	Acc. length	Accession
Pantoea agglomerans strain L15 plasmid pPagL15_1	Pantoea agglomerans	658	658	100%	0.0	100.00%	583567	CP034149.1
Pantoea agglomerans strain ASB05 plasmid pASB05p1	Pantoea agglomerans	656	656	100%	0.0	99.69%	563807	CP046723.1
Pantoea agglomerans strain AR5 plasmid pAR5_A	Pantoea agglomerans	656	656	100%	0.0	99.69%	555257	CP134754.1
Pantoea agglomerans strain AR8b plasmid pAR8b_A	Pantoea agglomerans	656	656	100%	0.0	99.69%	575199	CP134750.1
Pantoea agglomerans strain BH6c plasmid pBH6cv2_A	Pantoea agglomerans	656	656	100%	0.0	99.69%	569248	CP134745.1
Pantoea agglomerans strain FC61912-B plasmid pFC61912-B_A	Pantoea agglomerans	656	656	100%	0.0	99.69%	547579	CP134738.1
Pantoea agglomerans strain MMD61212-C plasmid pMMD61212-C	Pantoea agglomerans	656	656	100%	0.0	99.69%	612559	CP134734.1
Pantoea agglomerans strain ROTS050421 plasmid pROTS050421_A	Pantoea agglomerans	656	656	100%	0.0	99.69%	519021	CP134729.1
Pantoea agglomerans strain SUH1 plasmid pSUH1_A	Pantoea agglomerans	656	656	100%	0.0	99.69%	574725	CP134725.1
Pantoea agglomerans strain T88c plasmid pT88c_A	Pantoea agglomerans	656	656	100%	0.0	99.69%	555281	CP134720.1
Pantoea agglomerans strain NBBC-01 plasmid pNBBC01-1	Pantoea agglomerans	656	656	100%	0.0	99.69%	531583	CP099729.1
Pantoea agglomerans strain CB1 plasmid pCB1A	Pantoea agglomerans	656	656	100%	0.0	99.69%	569555	CP084198.1
Pantoea agglomerans strain AB378 plasmid unnamed1	Pantoea agglomerans	656	656	100%	0.0	99.69%	555125	CP113086.1
Pantoea agglomerans strain DAPP-PG734 genome assembly, plasmid: P1	Pantoea agglomerans	656	656	100%	0.0	99.69%	530328	OW970316.1
Pantoea agglomerans strain CHTF15 plasmid unnamed1	Pantoea agglomerans	656	656	100%	0.0	99.69%	514938	CP103402.1
Pantoea agglomerans strain CPHN 2 plasmid unnamed1	Pantoea agglomerans	656	656	100%	0.0	99.69%	583238	CP098412.1
Pantoea agglomerans strain Pa58 plasmid p1	Pantoea agglomerans	656	656	100%	0.0	99.69%	554101	CP091097.1
Pantoea agglomerans strain PSV1-7 plasmid unnamed1	Pantoea agglomerans	656	656	100%	0.0	99.69%	621667	CP091190.1
Pantoea agglomerans strain DBM 3797 plasmid pPA_DBM3797_1	Pantoea agglomerans	656	656	100%	0.0	99.69%	555522	CP086134.1
Pantoea agglomerans strain FDAARGOS 1447 plasmid unnamed2	Pantoea agglomerans	656	656	100%	0.0	99.69%	511735	CP077368.1
Pantoea agglomerans strain AR1a plasmid pAR1aA	Pantoea agglomerans	656	656	100%	0.0	99.69%	557771	CP059091.1
Pantoea agglomerans strain UAEU18 plasmid unnamed1	Pantoea agglomerans	655	655	100%	0.0	99.38%	513383	CP048034.1
Pantoea agglomerans strain TH81 plasmid unnamed1	Pantoea agglomerans	654	654	99%	0.0	99.69%	520959	CP031650.1
Pantoea agglomerans strain CFSAN047153 plasmid pCFSAN047153_1	Pantoea agglomerans	654	654	100%	0.0	99.38%	613013	CP034470.1
Pantoea agglomerans strain CFSAN047154 plasmid pCFSAN047154_1	Pantoea agglomerans	654	654	100%	0.0	99.38%	613013	CP034475.1
Pantoea agglomerans strain C410P1 plasmid unnamed1	Pantoea agglomerans	654	654	100%	0.0	99.38%	543504	CP016890.1
Pantoea agglomerans strain AR24 plasmid pAR24_A	Pantoea agglomerans	654	654	100%	0.0	99.38%	537957	CP134758.1
Pantoea agglomerans strain ZJU23 plasmid unnamed3	Pantoea agglomerans	654	654	100%	0.0	99.38%	567588	CP068443.1
Pantoea agglomerans strain AJ2b plasmid pAJ2b_A	Pantoea agglomerans	653	653	100%	0.0	99.38%	571710	CP134762.1
Pantoea agglomerans pv. gypsophilae strain 824-1 plasmid pPAG02	Pantoea agglomerans pv. gypsophilae	653	653	100%	0.0	99.38%	582658	CP122321.1
Pantoea agglomerans pv. betae strain 4188 plasmid pPAB02	Pantoea agglomerans pv. betae	652	652	100%	0.0	99.07%	541337	CP122325.1
Pantoea agglomerans strain 1.2.4 plasmid unnamed	Pantoea agglomerans	651	651	100%	0.0	99.07%	598453	CP134150.1
Pantoea vagans strain PV989 plasmid pPV989-508	Pantoea vagans	647	647	100%	0.0	97.52%	507680	CP028350.1
Pantoea vagans C9-1 plasmid pPag3	Pantoea vagans C9-1	645	645	100%	0.0	97.21%	529676	CP001895.1
Pantoea vagans strain LMG 24199 plasmid pVag1	Pantoea vagans	644	644	100%	0.0	96.90%	559692	CP038854.1
Pantoea vagans strain FDAARGOS_160 plasmid unnamed2	Pantoea vagans	644	644	100%	0.0	97.21%	470309	CP014127.2
Pantoea alfalfae strain CQ10 plasmid p1_CQ10	Pantoea alfalfae	644	644	100%	0.0	97.21%	235469	CP082293.1
Pantoea agglomerans strain FL1 plasmid unnamed1	Pantoea agglomerans	644	644	100%	0.0	97.21%	504646	CP126682.1
Pantoea sp. Lij88 plasmid unnamed1	Pantoea sp. Lij88	637	637	99%	0.0	96.58%	634148	CP118267.1
Pantoea vagans strain FBS135 plasmid pPant1	Pantoea vagans	632	707	100%	0.0	95.98%	526773	CP022517.1
Pantoea jilinensis strain D25 plasmid plas2	Pantoea jilinensis	632	706	100%	0.0	95.98%	483571	CP077748.1
Pantoea sp. MT58 plasmid unnamed1	Pantoea sp. MT58	632	707	100%	0.0	95.98%	522095	CP061084.1
Pantoea eucalypti strain LMG 24197 plasmid pEuc1	Pantoea eucalypti	630	707	100%	0.0	95.67%	529303	CP045721.1
Pantoea deleyi strain LMG24200 plasmid Plas2	Pantoea deleyi	625	625	100%	0.0	93.81%	450383	CP071407.1
Pantoea anthophila strain CL1 plasmid unnamed1	Pantoea anthophila	619	619	100%	0.0	93.19%	454539	CP110471.1
Pantoea agglomerans strain HJS002 plasmid unnamed1	Pantoea agglomerans	617	617	100%	0.0	92.57%	548340	CP090208.1
Pantoea ananatis strain YJ76 plasmid p_unamed1 sequenced	Pantoea ananatis	573	573	100%	0.0	86.38%	328324	CP022429.1
Pantoea ananatis strain JBR-LB3-16 plasmid unnamed	Pantoea ananatis	573	573	100%	0.0	86.38%	338834	CP090357.1
Pantoea ananatis strain R100 plasmid	Pantoea ananatis	572	572	100%	0.0	86.07%	331058	CP014208.1
Pantoea ananatis strain NN08200 plasmid unnamed2	Pantoea ananatis	572	572	100%	0.0	86.38%	307670	CP035036.1
Pantoea ananatis strain SGAir0210 plasmid pSGAir0210_2	Pantoea ananatis	572	572	100%	0.0	86.38%	304029	CP028034.2
Pantoea ananatis strain JT8-6 plasmid unnamed	Pantoea ananatis	572	572	100%	0.0	86.38%	272048	CP099543.1
Pantoea ananatis strain JT1-188 plasmid unnamed1	Pantoea ananatis	572	572	100%	0.0	86.38%	286165	CP099536.1
Pantoea ananatis strain Lstri plasmid pLs	Pantoea ananatis	572	572	100%	0.0	86.38%	267898	CP060819.1
Pantoea ananatis strain VY148 plasmid unnamed	Pantoea ananatis	572	572	100%	0.0	86.38%	302727	CP086008.1
Pantoea ananatis strain TZ39 plasmid plas2	Pantoea ananatis	572	572	100%	0.0	86.38%	276579	CP081344.1
Pantoea ananatis LMG 5342 plasmid pPANA10	Pantoea ananatis LMG 5342	572	572	100%	0.0	86.38%	302599	HE617161.1
Pantoea ananatis PA13 plasmid PAGR_p	Pantoea ananatis PA13	572	572	100%	0.0	86.38%	280753	CP003086.1
Pantoea ananatis AJ13355 plasmid pEA320 DNA	Pantoea ananatis AJ13355	572	572	100%	0.0	86.38%	321744	AP012033.1
Pantoea ananatis LMG 20103, complete genome	Pantoea ananatis LMG 20103	572	572	100%	0.0	86.38%	4703373	CP001875.2
Pantoea ananatis strain FDAARGOS_680 plasmid unnamed1	Pantoea ananatis	571	571	100%	0.0	86.38%	283516	CP054909.1
Pantoea ananatis strain PNA 97-1R plasmid pPNA97-1RA	Pantoea ananatis	571	571	100%	0.0	86.07%	273809	CP020944.2
Pantoea ananatis FU-01 plasmid pPaFU01 DNA	Pantoea ananatis	571	571	100%	0.0	86.07%	281268	AP019754.1
Pantoea ananatis strain OC5a plasmid pOC5aA	Pantoea ananatis	570	570	100%	0.0	86.07%	305982	CP059083.1

Description	Scientific name	Max score	Total score	Query cove-rage	E -value	Identity (%)	Acc. length	Accession
Pantoea stewartii strain ZJ-FGZX1 plasmid unnamed1	Pantoea stewartii	562	562	100%	0.0	84.52%	326337	CP049116.1
Pantoea stewartii subsp. stewartii DC283 plasmid pDSJ10	Pantoea stewartii subsp. stewartii DC283	562	562	100%	0.0	84.52%	304641	CP017591.1
Pantoea stewartii strain HR3-48 plasmid unnamed	Pantoea stewartii	561	561	100%	0.0	84.52%	333832	CP099541.1
Pantoea stewartii isolate RON18713 plasmid pPSbnut	Pantoea stewartii	560	560	100%	0.0	84.52%	262265	CP116286.1
Pantoea phytobeneficialis strain MSR2 plasmid pMSR2A	Pantoea phytobeneficialis	541	541	100%	6e-175	78.95%	656860	CP024637.1
Pantoea cypripedii strain NE1 plasmid pNE1A	Pantoea cypripedii	540	540	100%	8e-175	79.26%	673450	CP024769.1
Pantoea sp. At-9b plasmid pPAT9B01	Pantoea sp. At-9b	538	538	100%	4e-174	78.95%	793953	CP002434.1
Pantoea sp. SOD02 plasmid pSOD02	Pantoea sp. SOD02	536	536	100%	4e-173	79.26%	926844	CP102605.1
Pantoea sp. SO10 plasmid unnamed1	Pantoea sp. SO10	535	535	99%	4e-173	79.50%	744154	CP040096.1
Pantoea sp. X85 plasmid unnamed	Pantoea sp. X85	535	535	100%	4e-173	79.26%	771939	CP121109.1
Pantoea sp. SS70 plasmid unnamed1	Pantoea sp. SS70	535	535	100%	4e-173	79.26%	779368	CP117200.1
Pantoea agglomerans strain 33.1 plasmid p33.1_1	Pantoea agglomerans	535	535	99%	5e-173	78.57%	527897	CP083808.1
Pantoea piersonii strain URMC-2103A041 plasmid p527820	Pantoea piersonii	533	533	99%	3e-172	77.95%	527820	CP115896.1
Pantoea piersonii strain GABEKP28 plasmid pGABEKP28_1	Pantoea piersonii	531	531	99%	1e-171	77.64%	513647	CP104759.1
Pantoea dispersa strain ESL4 plasmid pESL4.1	Pantoea dispersa	529	529	100%	9e-171	76.78%	690879	CP109854.1
Pantoea dispersa strain JL.02bL plasmid pJL.02bL	Pantoea dispersa	529	529	100%	9e-171	76.78%	696693	CP107574.1
Pantoea dispersa strain Lsch plasmid unnamed	Pantoea dispersa	529	529	100%	1e-170	76.47%	689940	CP082347.1
Pantoea dispersa strain VWJL.P1 plasmid pVWJL.P1	Pantoea dispersa	528	528	100%	1e-170	76.78%	708540	CP118630.1
Pantoea dispersa strain YSD_J2 plasmid unnamed	Pantoea dispersa	528	528	100%	1e-170	76.78%	710238	CP074351.1
Pantoea dispersa strain ML.8a3 plasmid pML.8a3	Pantoea dispersa	526	526	100%	6e-170	76.16%	742161	CP106661.1
Pantoea dispersa strain AHKW2b plasmid unnamed	Pantoea dispersa	526	526	100%	1e-169	75.85%	653898	CP082342.1
Pantoea sp. PSNIH1 plasmid pPSP-3a9	Pantoea sp. PSNIH1	486	486	99%	5e-156	72.59%	329383	CP010326.1
Pantoea eucrina strain XL123 plasmid unnamed2	Pantoea eucrina	486	486	99%	5e-156	72.59%	342726	CP083450.1

2.2. Supplementary Figures

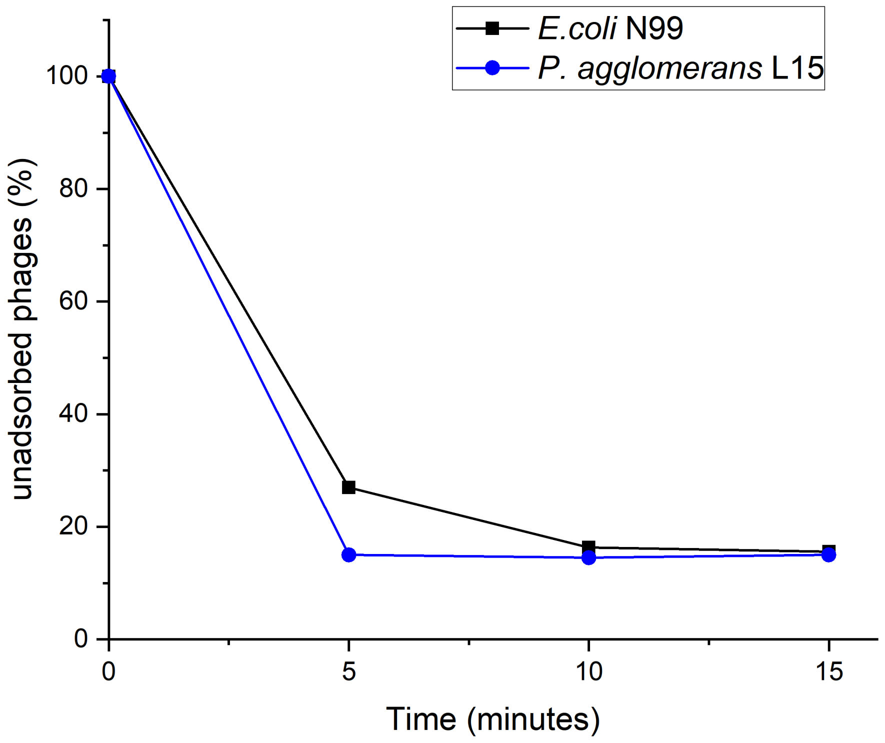

Supplementary Figure 1. Adsorption rate of bacteriophage P1 *c1-100* Tn*9* to *E. coli* N99 cells and *P. agglomerans* L15 cells. Cultures for the measurements were grown at 30° C.

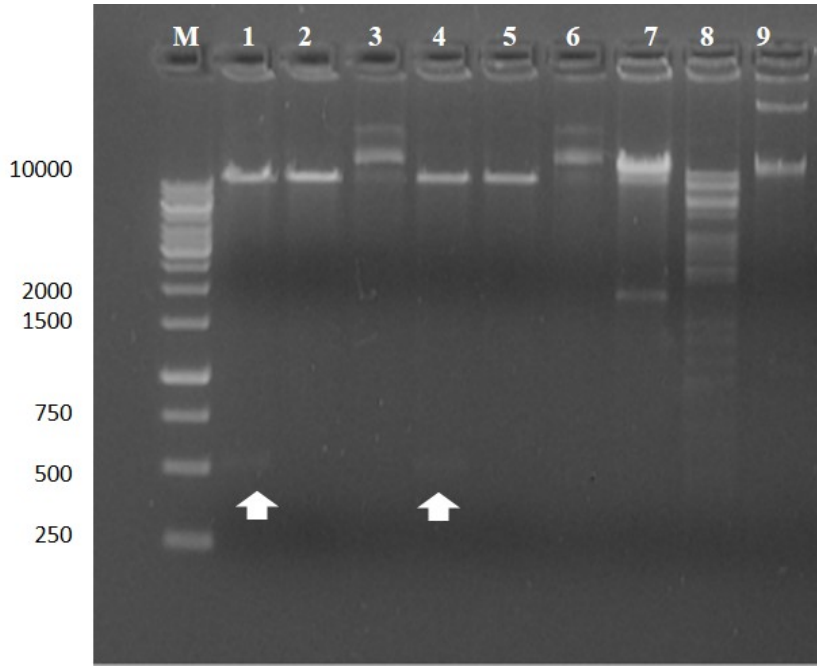

Supplementary Figure 2. Verification of the presence of plasmid pKGI5 in the transductants of *E. coli* C600 cells. Plasmid DNA isolated from the transductants obtained as resistant to tetracycline and sensitive to chloramphenicol (lane 1 - 6) or resistant to tetracycline and chloramphenicol (lane 7 - 9) after treatment with P1 *c1-100* Tn*9* induced from tetracycline and chloramphenicol resistant lysogens of the *E. coli* C600/pKGI5 strain was digested with PstI (lanes 1, 4, 7) or EcoRV (lanes 2, 5, 8) and separated in 1% agarose gel. Lanes 3, 6, and 9 represented undigested DNA. GeneRuler™ 1 kb DNA ladder (Thermo Fisher Scientific) was used as a DNA size marker (M). As expected PstI cleaved the plasmids obtained from the $Tc^{R}Cm^{S}$ clones into the ca. 500 bp fragment (indicated by an arrow) representing the cloned fragment of *phddoc* operon that is flanked by the two PstI recognition sites in pKGI5, and over 10 kB fragment representing the rest of pKGI5, while EcoRV which has one recognition site in pKGI5 linearized the plasmid. The pattern of PstI and EcoRV cleavage of plasmid DNA from $Tc^{R}Cm^{R}$ clones was more complex and consistent with the expected cleavage pattern of P1-pKGI5 hybrids, which have three PstI recognition sites (one in the IS*1* sequence of P1 and two in the pKGI5) and several EcoRV sites (31 in the P1 derived DNA and one in pKGI5-derived DNA).

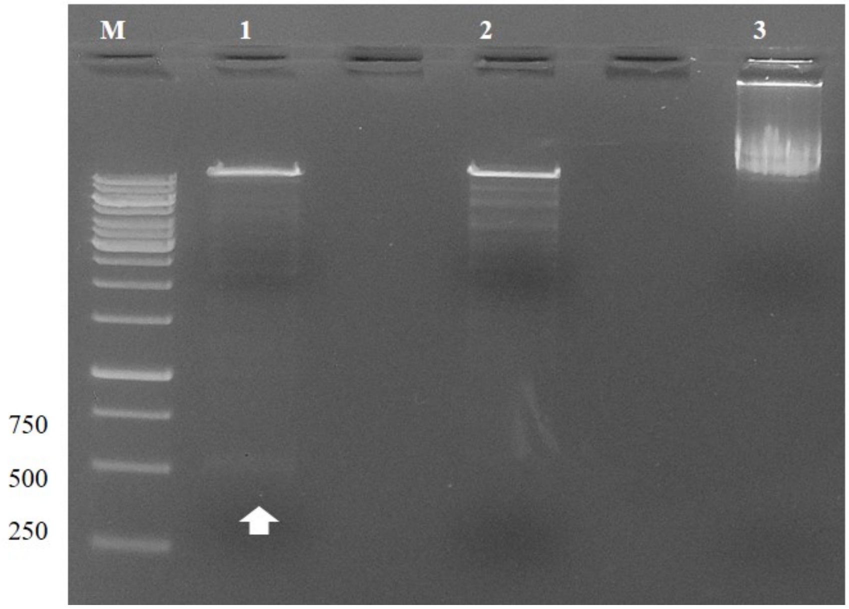

Supplementary Figure 3. Verification of the presence of plasmid pKGI5 in the transductants of *P. agglomerans* L15 cells. Plasmid DNA isolated from the transductants obtained as resistant to tetracycline and sensitive to chloramphenicol after infection with hybrid phage P1 *c1-100* Tn*9*-pKGI5 induced from the tetracycline and chloramphenicol resistant lysogen of the *E. coli* C600 strain was digested with PstI (lane 1) or EcoRV (lane 2), and separated in 1% agarose gel. As expected PstI cleaved the plasmid obtained from the $Tc^{R}Cm^{S}$ clones into the ca. 500 bp fragment (indicated by an arrow) representing the cloned fragment of *phddoc* operon, and over 10 kB fragment representing the rest of pKGI5, while EcoRV which has one recognition site in pKGI5 linearized the plasmid. The background of smaller intensity bands in addition to bands representing digestion products of pKGI5 likely represents digested DNA of native *P. agglomerans* L15 plasmids. Lane 3 represents undigested DNA. The presence of native plasmids in the L15 strain is reflected by the smear over the band representing the 10 kb marker fragment. GeneRuler^{TM} 1 kb DNA ladder (Thermo Fisher Scientific, Gdańsk) was used as a DNA size marker (M). The ca. 500 bp Pst-PstI fragment of pKGI5 representing the cloned *phddoc* region is indicated by a arrow.

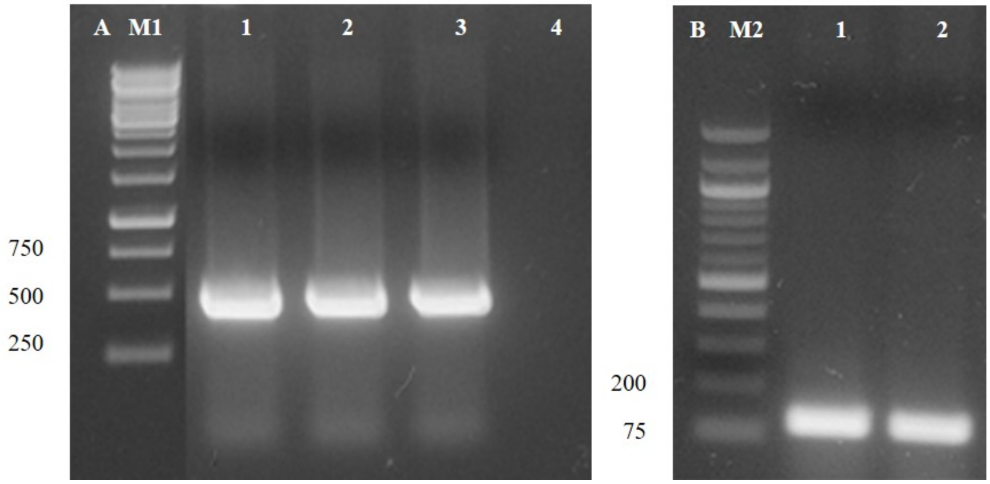

Supplementary Figure 4. Verification of the presence of plasmid pKGI5 in the transductants of *P. agglomerans* L15 cells. Plasmid DNA isolated from the transductants obtained as resistant to tetracycline and sensitive to chloramphenicol after infection with P1 *c1-100* Tn*9* induced from tetracycline and chloramphenicol resistant lysogens of *E. coli* C600/pKGI5 strain was used as a template in amplification with primers **(A)** OMLO755 and OMLO756 complementary to the flanking regions of P1 *phddoc* operon fragment cloned in pKGI5, and **(B)** trfAF and trfAR complementary to the region flanking the *trfA trfB* operon of RK2 in the pRK415 plasmid. The amplicons were separated in 1% agarose gel. Lanes 1 and 2 in each gel represent amplicons of plasmid fragments from the transductants. Lane A3 represents the control amplicon with P1 c1-100 Tn9 DNA as a template. Lane A4 represents no DNA control. GeneRuler™ 1 kb DNA ladder (Thermo Fisher Scientific) was used as a DNA size marker (M1); GeneRuler™ 1 kb Plus DNA ladder (Thermo Fisher Scientific) was used as a DNA size marker (M2).

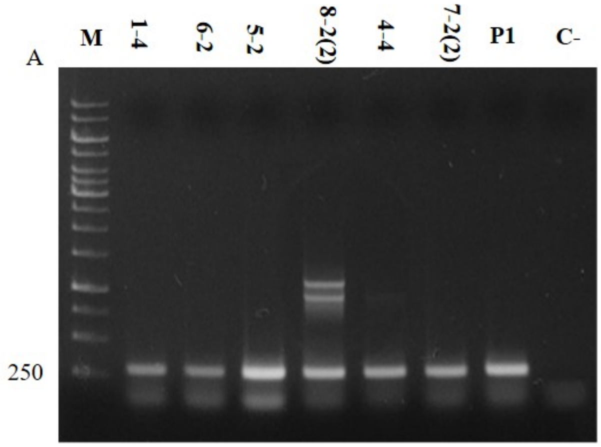

B

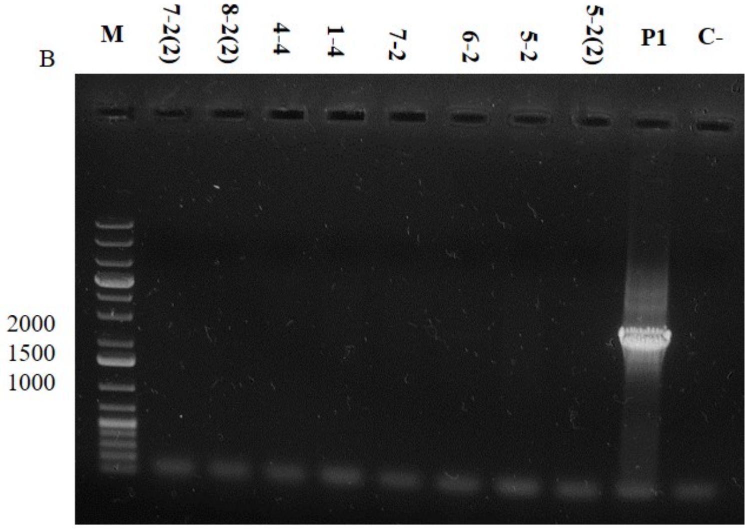

C

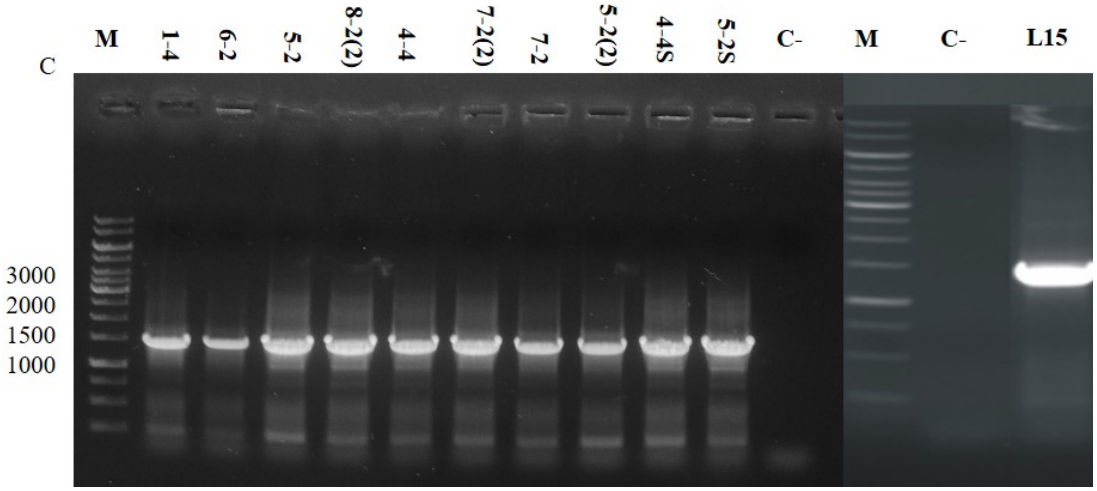

Supplementary Figure 5. Detection of P1 *c1-100* Tn*9* genome components in certain clones of P1 *c1-100* Tn*9*-treated *P. agglomerans* L15 cells of stable chloramphenicol resistance phenotype. Cells that were used as a source of templates for colony PCR were taken from the tops of freshly passaged colonies grown on LA medium with chloramphenicol. **(A)** Amplicons obtained with the OMLO533 and OMLO534 primers for the detection of the Tn*9* *cat* gene. **(B)** Amplicons obtained with the OMLO554 and OMLO555 primers for the detection of P1 *par* operon. **(C)** Amplicons obtained with the OMLO531 and OMLO532 primers for the detection of *P. agglomerans gyrB* gene. Gel lanes representing amplicons obtained with P1 *c1-100* Tn*9* and *P. agglomerans* L15 DNA as a template are marked with P1 and L15, respectively. Lanes marked with C indicate control samples without template DNA. Gel lanes marked with M in (A) and (B) represent DNA size marker - GeneRuler™ 1 kb DNA ladder (Thermo Fisher Scientific); the one in (C) represents DNA size marker - GeneRuler™ 1 kb Plus DNA ladder (Thermo Fisher Scientific). Other gel lanes are marked with the numbers representing the designations of clones that served as sources of template DNA in amplification reactions.

A

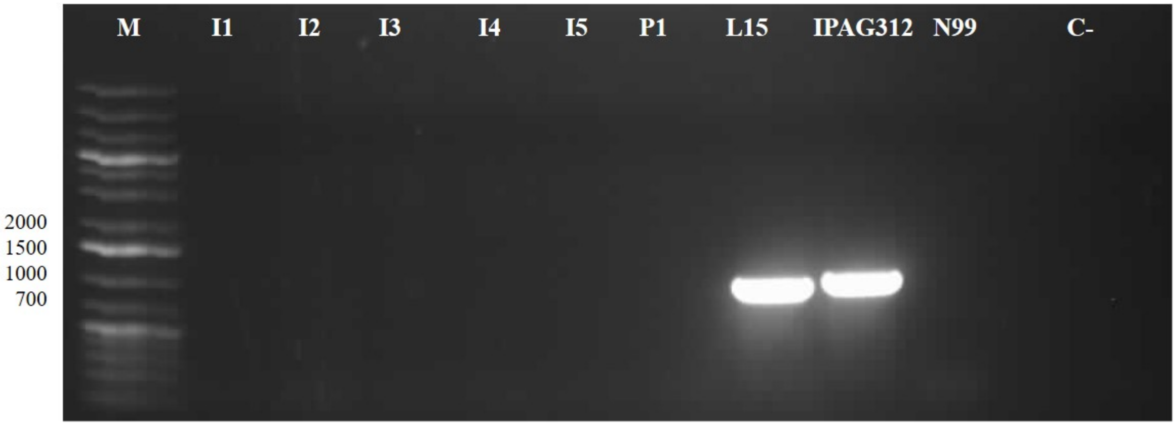

B

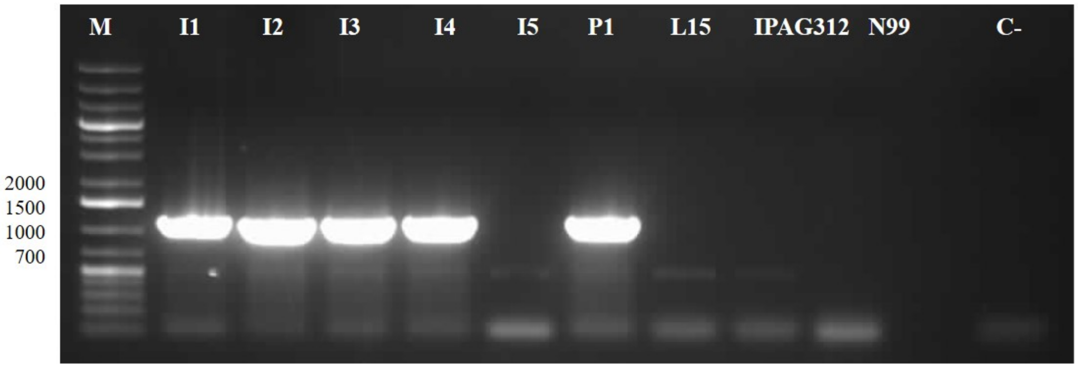

Supplementary Figure 6. Detection of plasmid pPagL15_3 DNA (A) and prophage P1 *c1-100* Tn*9* IS*1*::$ccdA_{pPagL15\_3}$ DNA (B) in the *P. agglomerans* IPAG312 cells of certain clones lysogenized with P1 *c1-100* Tn*9* IS*1*::$ccdA_{pPagL15\_3}$. and restreaked several times on M9 minimal solid medium with glucose, thiamine and chloramphenicol and LA medium with chloramphenicol. A and B show, respectively, amplicons obtained after colony PCR amplification with OMLO921 and OMLO922 specific for the *parB* gene of pPagL15_3 plasmid, and with OMLO919 and OMLO920 primers specific for the bacteriophage P1 *pdcB* gene. Gel lanes representing amplicons obtained with P1, and *P. agglomerans* L15 and IPAG312 DNA as a template are marked with P1, L15, and IPAG312, respectively. Lanes marked with N99 and C indicate control samples with nonspecific DNA and without template DNA, respectively. Gel lanes marked with M represent DNA size marker - GeneRuler^{TM} 1 kb Plus DNA ladder (Thermo Fisher Scientific). Other gel lanes (I1-I5) are marked with the numbers representing the designations of clones that served as sources of template DNA in amplification reactions.

A

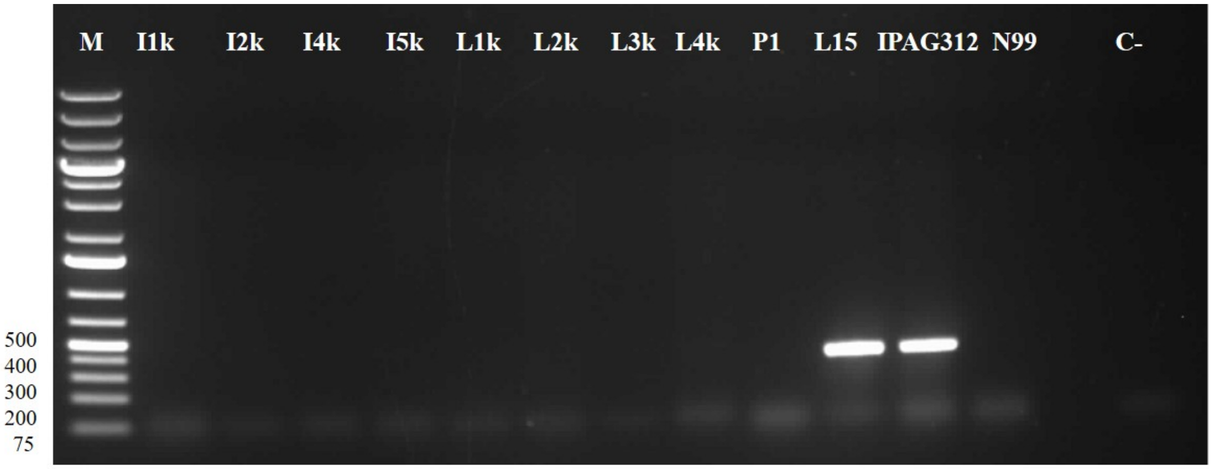

B

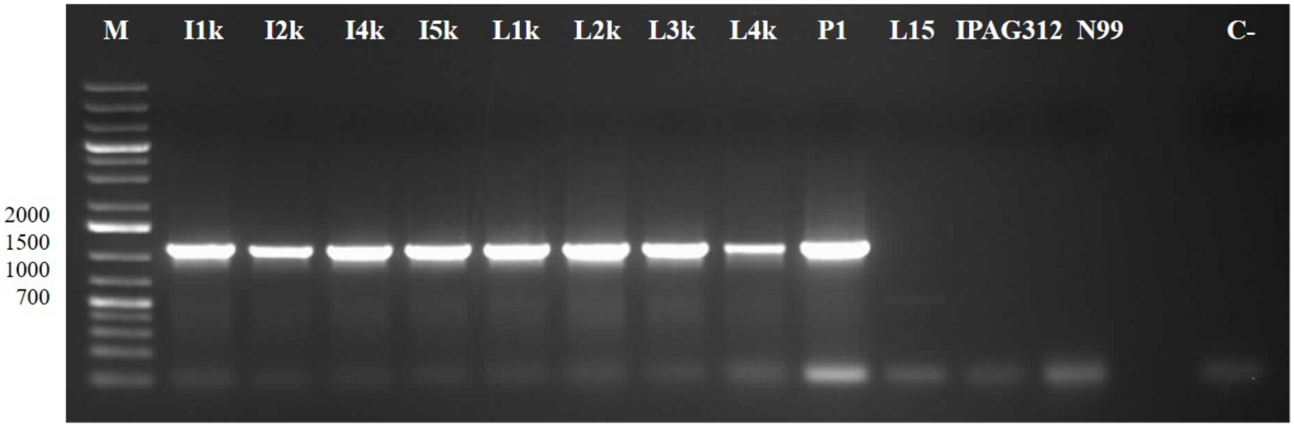

Supplementary Figure 7. Detection of plasmid pPagL15_3 DNA (A) and prophage P1 *c1-100* Tn*9* IS*1*::km^R DNA (B) in the *P. agglomerans* L15 and IPAG312 cells of certain clones lysogenized with P1 *c1-100* Tn*9* IS*1*::km^R, and restreaked several times on M9 minimal solid medium with glucose, thiamine and chloramphenicol and LA medium with chloramphenicol. A and B show, respectively, amplicons obtained after colony PCR amplification with OMLO934 and OMLO952 primers specific for the *ccdA* gene of pPagL15_3 plasmid, and with OMLO919 and OMLO920 primers specific for the bacteriophage P1 *pdcB* gene. Gel lanes representing amplicons obtained with P1, and *P. agglomerans* L15 and IPAG312 DNA as a template are marked with P1, L15, and IPAG312, respectively. Lanes marked with N99 and C indicate control samples with nonspecific DNA and without template DNA, respectively. Gel lanes marked with M represent DNA size marker - GeneRuler™ 1 kb Plus DNA ladder (Thermo Fisher Scientific). Other gel lanes, I1k-I5k, and L1k-l4k, are marked with the numbers representing the designations of clones that served as sources of template DNA in amplification reactions: the IPAG312 lysogens, and the L15 lysogens, respectively.

3 Supplementary References

Keen, N. T., Tamaki, S., Kobayashi, D., and Trollinger, D. (1988). Improved broad host- range plasmids for DNA cloning in gram-negative bacteria. *Gene* 70, 191–197. doi: 10.1016/0378-1119(88)90117-5.

Taylor, L. A., and Rose, R. E. (1988). A correction in the nucleotide sequence of the Tn*903* kanamycin resistance determinant in PUC4K. *Nucl. Acids Res.* 16, 358–358. doi: 10.1093/nar/16.1.358.

Vieira, J., and Messing, J. (1982). The pUC plasmids, an M13mp7-derived system for insertion mutagenesis and sequencing with synthetic universal primers. *Gene* 19, 259–268. doi: 10.1016/0378-1119(82)90015-4.
